# The internal cranial anatomy of *Romundina stellina* Ørvig, 1975 (Vertebrata, Placodermi, Acanthothoraci) and the origin of jawed vertebrates—Anatomical atlas of a primitive gnathostome

**DOI:** 10.1371/journal.pone.0171241

**Published:** 2017-02-07

**Authors:** Vincent Dupret, Sophie Sanchez, Daniel Goujet, Per Erik Ahlberg

**Affiliations:** 1 Science for Life Laboratory and Uppsala University, Department of Organismal Biology, Subdepartment of Evolution and Development, Norbyvägen, SE Uppsala, Sweden; 2 European Synchrotron Radiation Facility, Grenoble, France; 3 Centre de Recherche sur la Paléobiodiversité et les Paléoenvironnements (CR2P, UMR 7207), Sorbonne Universités, MNHN, CNRS, UPMC-Paris 6, Muséum National d’Histoire Naturelle, Paris, France; Institute of Botany, CHINA

## Abstract

Placoderms are considered as the first jawed vertebrates and constitute a paraphyletic group in the stem-gnathostome grade. The acanthothoracid placoderms are among the phylogenetically most basal and morphologically primitive gnathostomes, but their neurocranial anatomy is poorly understood. Here we present a near-complete three-dimensional skull of *Romundina stellina*, a small Early Devonian acanthothoracid from the Canadian Arctic Archipelago, scanned with propagation phase contrast microtomography at a 7.46 μm isotropic voxel size at the European Synchrotron Radiation Facility, Grenoble, France. This is the first model of an early gnathostome skull produced using this technique, and as such represents a major advance in objectivity compared to past descriptions of placoderm neurocrania on the basis of grinding series. Despite some loss of material along an oblique crack, most of the internal structures are remarkably preserved, and most of the missing structures can be reconstructed by symmetry. This virtual approach offers the possibility to connect with certainty all the external foramina to the blood and nerve canals and the central structures, and thus identify accurate homologies without destroying the specimen. The high level of detail enables description of the main arterial, venous and nerve canals of the skull, and other perichondrally ossified endocranial structures such as the palatoquadrate articulations, the endocranial cavity and the inner ear cavities. The braincase morphology appears less extreme than that of *Brindabellaspis*, and is in some respects more reminiscent of a basal arthrodire such as *Kujdanowiaspis*.

## Introduction

**Fig 1:**
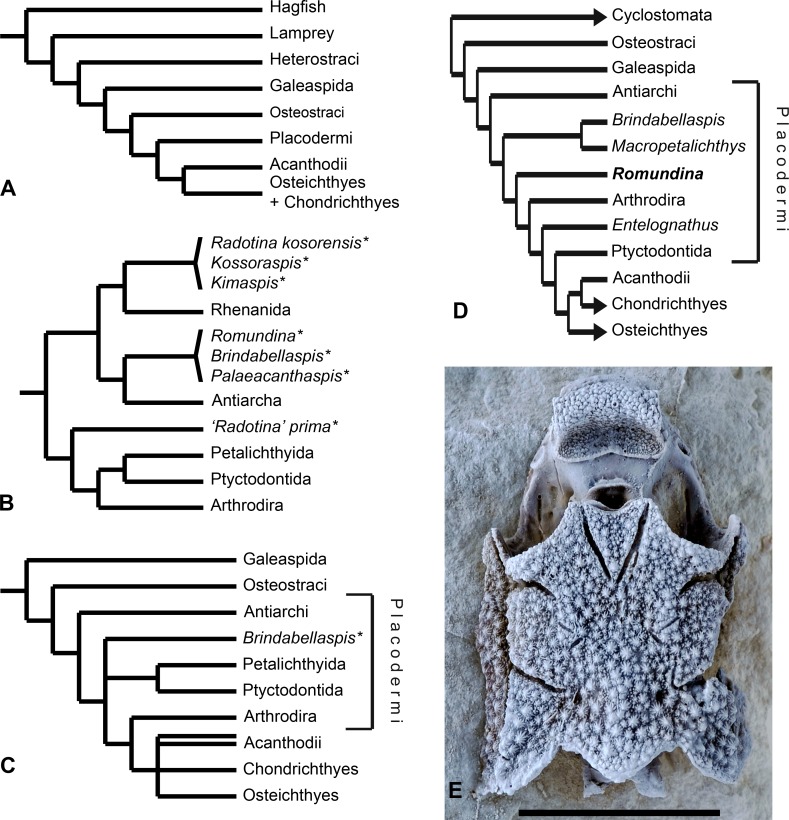
Vertebrate relationships and *Romundina*. A. General phylogenetic relationships among Vertebrata (modified after [5]:fig 9.1). B. Phylogenetic relationships among the Placodermi, resolving them as a monophyletic group, among which the Acanthothoraci (indicated by an asterisk) are not monophyletic (modified after [5]:fig 4.57). C. Phylogenetic relationships among Vertebrata with Placodermi resolved as a paraphyletic group within stem gnathostomes (modified after [1]). D. Phylogenetic relationships among Vertebrata with Placodermi resolved as a paraphyletic segment of the gnathostome stem group (modified after [6]). E. Skull of *Romundina stellina* [7] in dorsal view (specimen MNHN.F.CPW1; scale bar: 1 cm).

The Placodermi, armoured jawed fishes of the Silurian to Devonian periods (430–360 million years old), are an entirely extinct major group of gnathostomes (jawed vertebrates). Conventionally they have been regarded as a clade and placed in the top of the gnathostome stem group as the sister group to the gnathostome crown (Fig 1A). However, recent phylogenetic re-evaluations of deep gnathostome relationships [1–3] have suggested that placoderms could be paraphyletic relative to crown gnathostomes (Fig 1B–1D). This implies that placoderms may be uniquely informative about the evolution of gnathostome body architecture, the single most dramatic morphological transformation in vertebrate evolution and a key step in our own ancestry. In particular, the earliest and most primitive placoderms have great potential to illuminate the evolution of jawed vertebrate traits. The most recent analysis resolves placoderms as a clade [4].

Here we present the cranial anatomy of *Romundina*, an Early Devonian (415 million years old) placoderm that has previously been assigned to the “Acanthothoraci”, a probably non-monophyletic grade taxon of primitive placoderms. The description is based on a three-dimensionally preserved skull that has been imaged by means of propagation phase contrast tomography using synchrotron radiation, allowing us to reconstruct the virtually complete internal architecture of the specimens in three dimensions with unprecedented resolution and accuracy. This is the earliest and phylogenetically most basal jawed vertebrate cranium to be investigated in this manner.

## Material and methods

### Systematic paleontology

Class Placodermi [8]

Order Acanthothoraci [9]

Family Palaeacanthaspididae [10]

Genus *Romundina* [7]

Species *Romundina stellina* [7]

Specimen MNHN.F.CPW1

Figs 1–16

**Fig 2:**
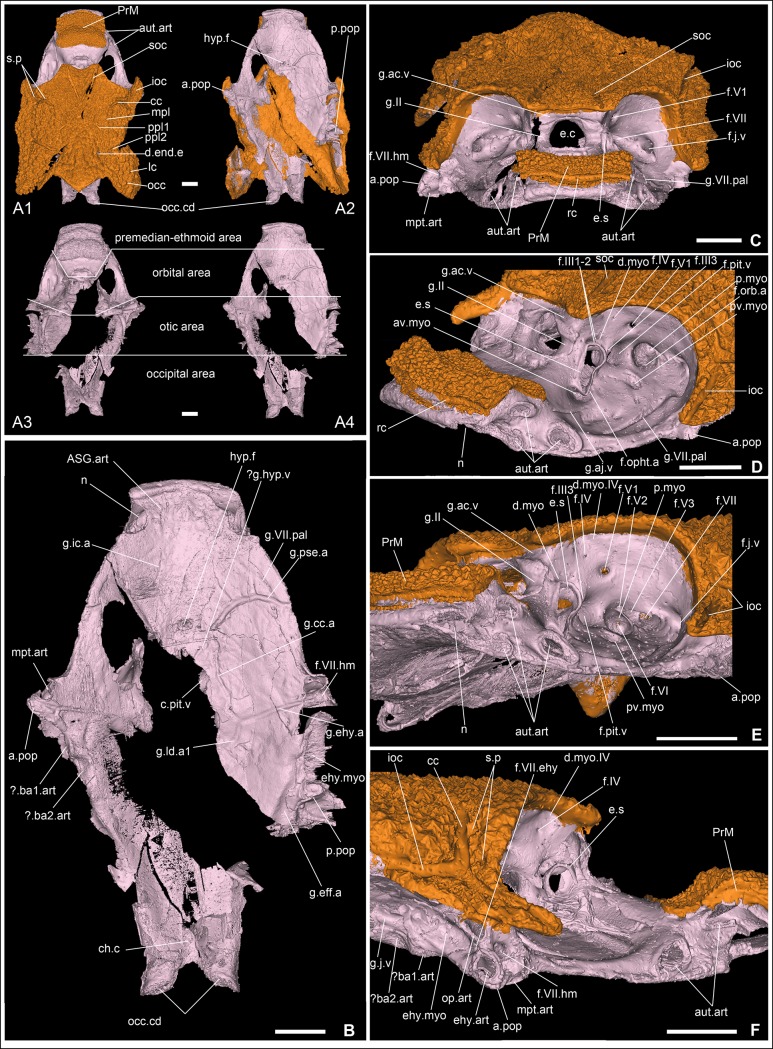
Skull roof and external aspect of the braincase of *Romundina stellina* [7], specimen MNHN.F.CPW1. A1-2. Skull roof (orange) and perichondral bone cover of the braincase (EPB in the text; light pink) in dorsal (A1) and ventral (A2) views. A3-4. Perichondral bone cover of the braincase in dorsal (A3) and ventral views (A4), with emphasis on the different areas of the neurocranium. The perichondral bone underlying the paranuchal plates has been removed. Notice the oblique crack (that also provoked the collapse of the medial wall of the right orbit), and the incompleteness of the braincase floor. B. Neurocranium in ventral view (the perichondral bone underlying the paranuchal plates has been removed). C. Skull roof and neurocranium (premedian-ethmoid and orbital areas) in anterior view, slightly dorsal (the lateral semicircular canal is horizontal). D. Skull roof and neurocranium (premedian-ethmoid and orbital areas) in left anterodorsolateral view. E. Skull roof and neurocranium (premedian-ethmoid and orbital areas) in left anterolateral view. F. Skull roof and neurocranium (premedian-ethmoid, orbital and partly otic areas) in right lateral view. Scale bars are 2 mm in length.

**Fig 3:**
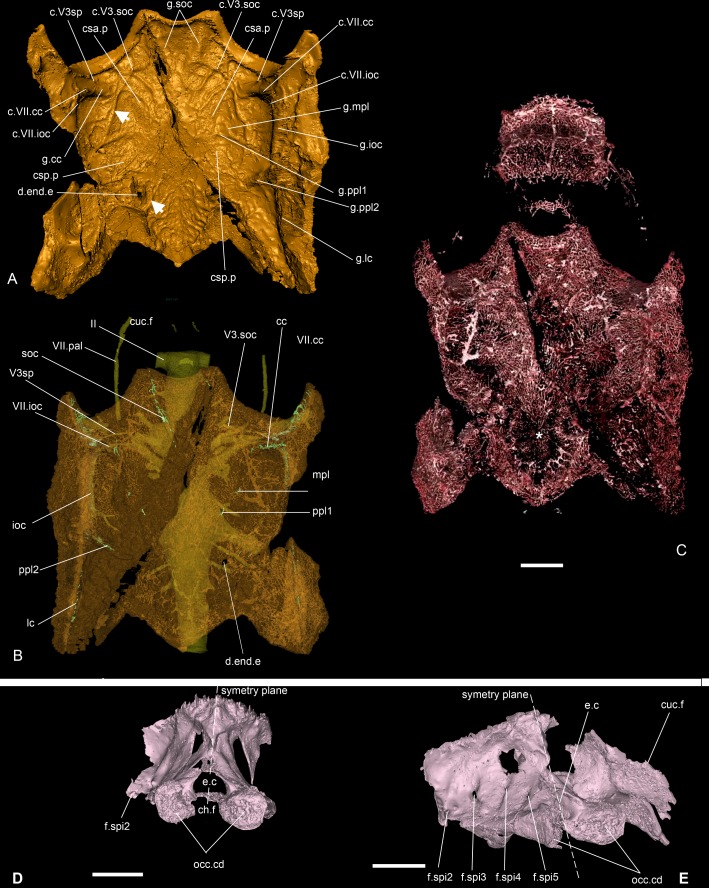
Dermal skull roof and blood vessels of *Romundina stellina* [7], specimen MNHN.F.CPW1. and occipital area of the braincase. A. Dermal skull roof in ventral view. B. Semitransparent dermal skull roof in dorsal view, showing the small canals transmitting nerve branches to the lateral line grooves (green) and the outline of the underlying cranial cavity. C. Vasculature of the skull roof in dorsal view. Scale bars are 2 mm in length. D, E. Occipital area of the neurocranium in posterior (C) and left posterolateral (D) views. In order to clarify the figure, the perichondral bone layer under the paranuchal plates (except for Fig 3E, right side) and the parts anterior to the occipital area have been obliterated. White arrows indicate vascular canals at the boundary between dermal and perichondral bone layers; asterisk indicates radiating centre of nuchal plate. Scale bars are 2 mm in length.

**Fig 4:**
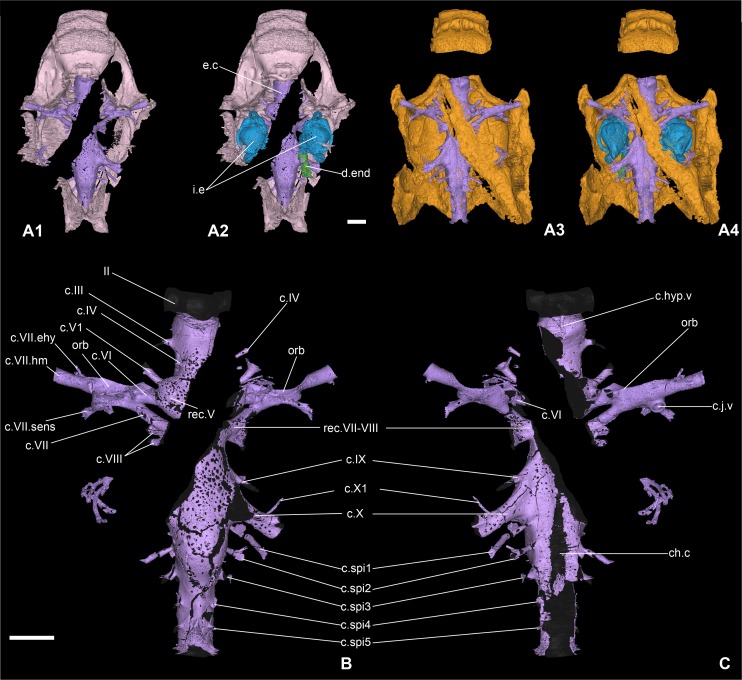
Endocranial cavity of the braincase of *Romundina stellina* [7], specimen MNHN.F.CPW1. A1-2. Perichondral bone of the neurocranium and the endocranial cavity (A1) with inner ears and right endolymphatic duct (A2) in dorsal view; the perichondral bone underlying the paranuchal plates has been digitally removed for clarity. A3-4. Dermal bone of the skull roof and perichondral bone of the endocranial cavity (A3) with the inner ears and right endolymphatic duct (A4) in ventral view. B-C. Endocranial cavity and cranial nerve canals in dorsal (B) and ventral (C) views. The endocranial cavity has been digitally filled in black in order to clarify the lace pattern of the perichondral bone (otherwise obscured by the visual interaction between the dorsal and ventral sides of the cavity). Scale bars are 2 mm in length.

**Fig 5:**
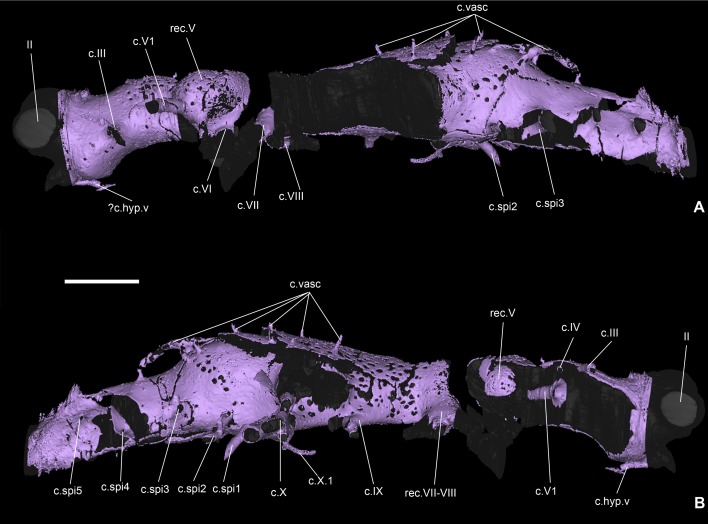
Endocranial cavity of the braincase of *Romundina stellina* [7], specimen MNHN.F.CPW1 (continued). Endocranial cavity in left (A) and right (B) lateral views. The endocranial cavity has been digitally filled in black in order to clarify the lace pattern of the perichondral bone. The lateral parts of the endocranial cavity (i.e. cranial nerve canals and their dorsal extensions) have been digitally removed for clarity. Scale bars are 2 mm in length.

**Fig 6:**
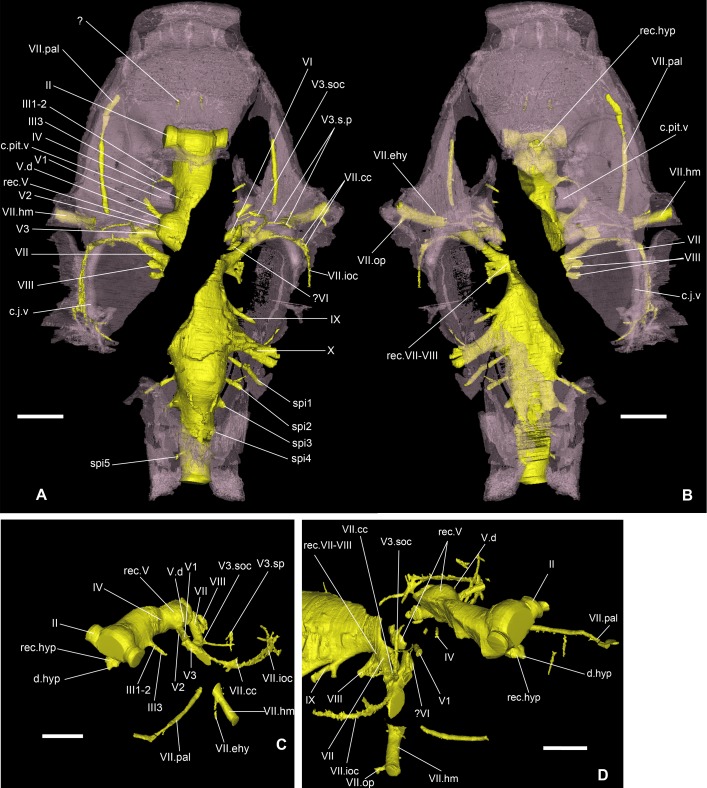
Nervous system of *Romundina stellina* [7], specimen MNHN.F.CPW1. Filled endocranial cavity and nerve canals and grooves (yellow); perichondral bone in transparent pink. A. Dorsal view. B. Ventral view. C. Left oblique anterolateral slightly dorsal view (only portion anterior to the oblique crack is presented). D. Right oblique anterolateral slightly dorsal view. Scale bars are 2 mm in length.

**Fig 7:**
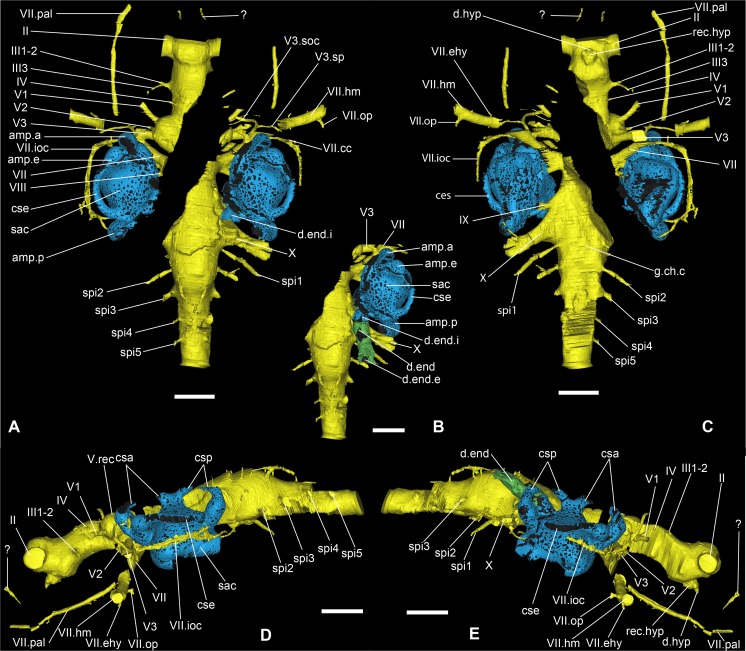
Nervous system of *Romundina stellina* [7], specimen MNHN.F.CPW1 (continued). Filled endocranial cavity and nerve canals and grooves, in relation with the inner ear organ. A. Dorsal view. B. Dorsal view with endolymphatic duct visible. C. Central view. D. Left lateral view. E. Right lateral view. Scale bars are 2 mm in length.

**Fig 8:**
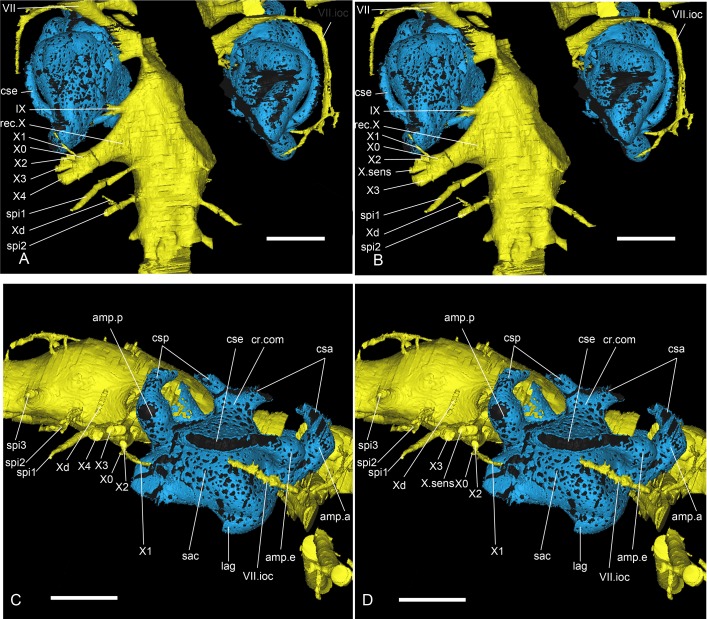
Different interpretations of the vagal nerve area of *Romundina stellina* [7], specimen MNHN.F.CPW1. (A, B) Ventral views and (C, D) Right lateral views. Scale bars are 2 mm in length.

**Fig 9:**
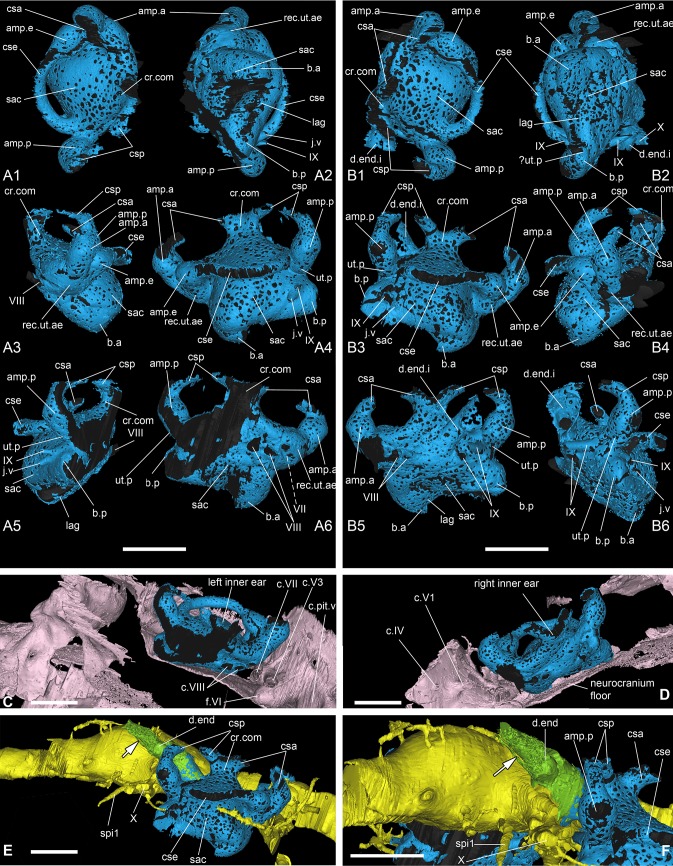
Inner ear and endolymphatic duct of *Romundina stellina* [7], specimen MNHN.F.CPW1. A: Left inner ear in dorsal (A1), ventral (A2), anterior (A3), left lateral (A4), posterior (A5) and right medial view (A6). B: Right inner ear in dorsal (B1), ventral (B2), right lateral (B3), anterior (B4), left medial view (B5) and posterior (B6). C. Left internal view from the inside revealing the slight contact between the left inner ear and the ventral wall of the neurocranium. D. Right internal view from the inside revealing the absence of contact between the right inner ear and the ventral wall of the neurocranium. E. Right lateral view of the filled endocranial cavity and the right inner ear and endolymphatic duct, with associated dermal bone vascularization. F. Right posterolateral view of the filled endocranial cavity and the right inner ear and endolymphatic duct, with associated dermal bone vascularization. A-D: Scale bars are 2 mm in length. For clarity, the inner ears have been filled in black.

**Fig 10:**
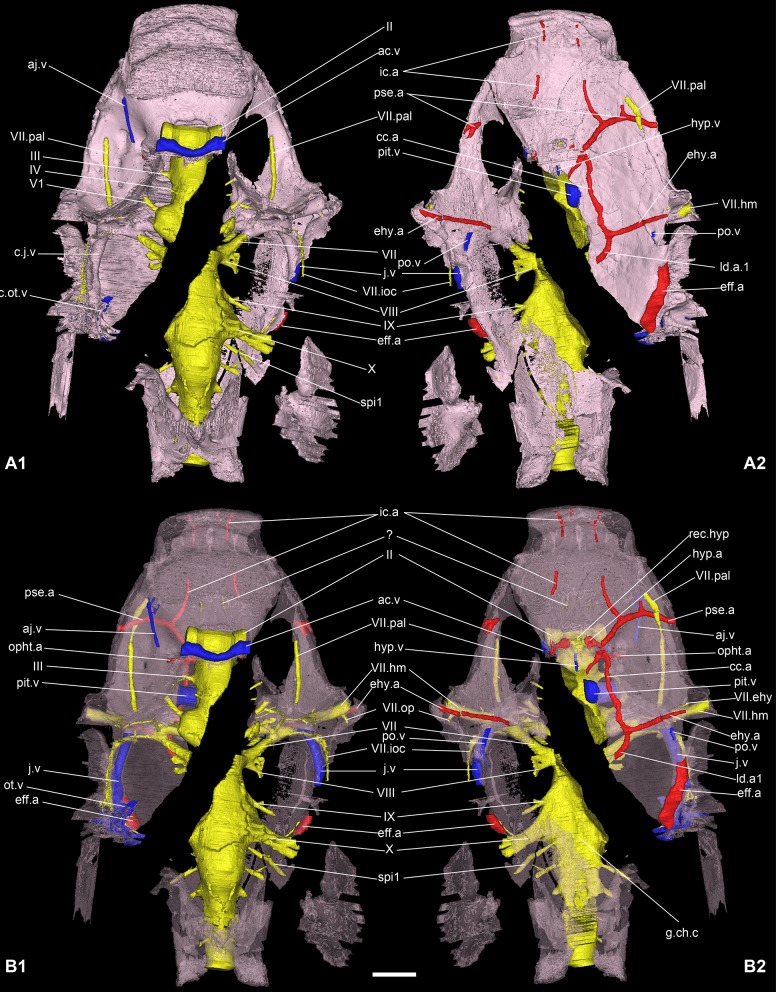
Main cranial vascularization of *Romundina stellina* [7], specimen MNHN.F.CPW1. The vasculature has been reconstructed on the basis of preserved grooves and canals in the braincase. Veins (in blue) and arteries (in red) are presented in relation to the central nervous system (in yellow) and the perichondral bone of the braincase (in pink; opaque in A; semitransparent in B) in dorsal (A1, B1) and ventral views (A2, B2). Scale bars are 2 mm in length.

**Fig 11:**
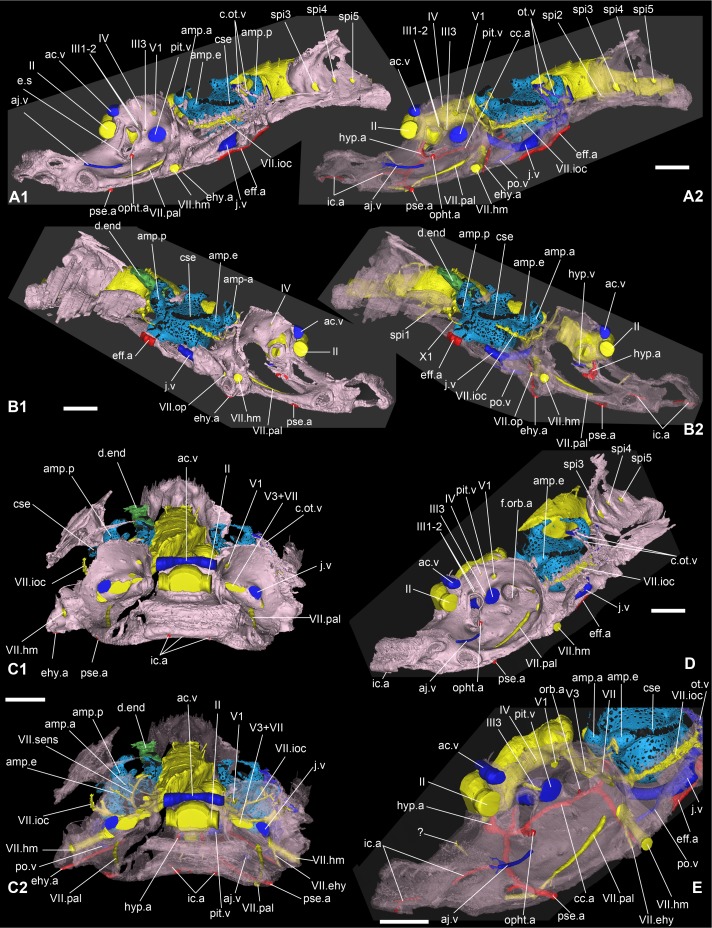
Main cranial vascularization of *Romundina stellina* [7], specimen MNHN.F.CPW1 (continued). Veins (in blue) and arteries (in red) are presented in relation to the central nervous system (in yellow) and the perichondral bone of the braincase (in pink; opaque in 1; semitransparent in 2) in left lateral (A), right lateral (B), anterior (C) and oblique anterodorsolateral view (D), with a special emphasis on the orbit area (E). Scale bars are 2 mm in length. A, B,D-E show an opaque wall in the symmetry plane for clarity (the other half of the specimen is not visible).

**Fig 12:**
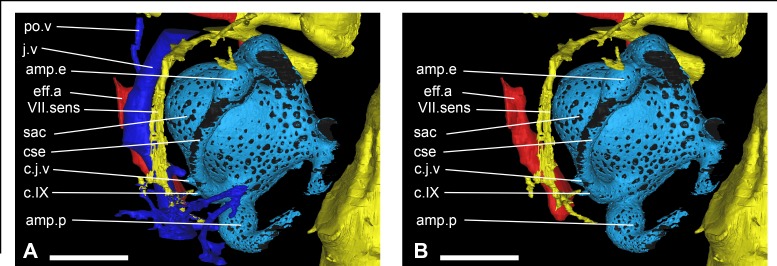
Main vascularization in the otic area of *Romundina stellina* [7], specimen MNHN.F.CPW1. Veins (in blue) and arteries (in red) are presented in relation to the central nervous system (in yellow) in the left otic area (light blue), in slightly oblique dorsal view (A with veins; B without veins for clarity). Scale bars are 2 mm in length.

**Fig 13:**
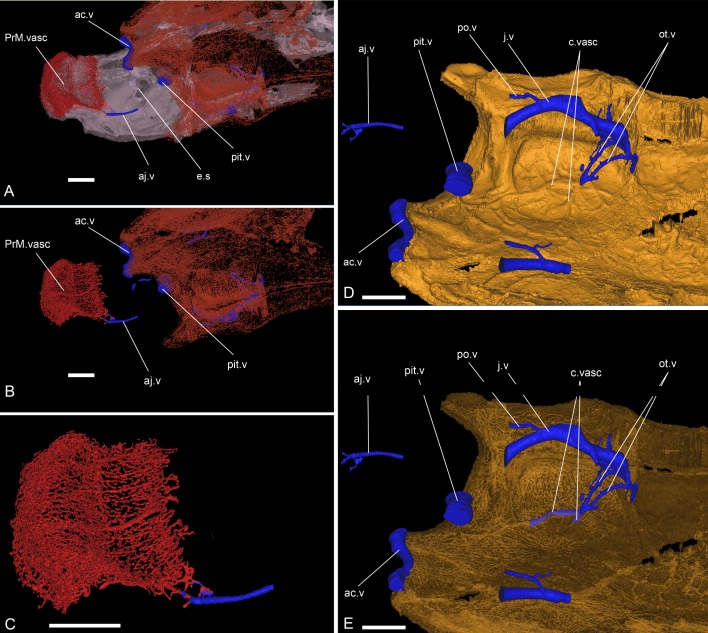
Relationships between the dermal bone vessels and the intracranial vascularization and innervation of *Romundina stellina* [7], specimen MNHN.F.CPW1. Relationships between the dermal bone vasculature and the intracranial vascularization and innervation. A. Connection between the vasculature of the premedian plate and the anterior jugular vein with external perichondral bone semitransparent, or B. fully transparent. C. Close up emphasizing the connection. D-E. Oblique ventral view of the skull roof (opaque in D; semi-transparent in E) showing the connection between the vertical, curved otic veins and the skull roof vasculature and possibly the sensory line system. Scale bars are 2 mm in length.

**Fig 14:**
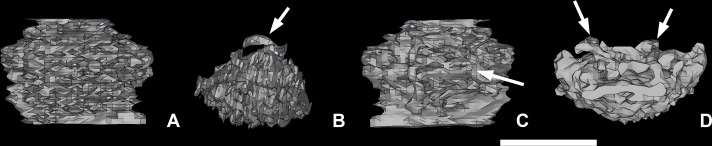
Possible parasphenoid of *Romundina stellina* [7], specimen MNHN.F.CPW1. Vascular mesh in ventral (A), left lateral (B), dorsal (C) and anterior (D) views. Arrows indicate canals connected to although not incorporated in the mesh. Scale bar is 0.5 mm in length.

**Fig 15:**
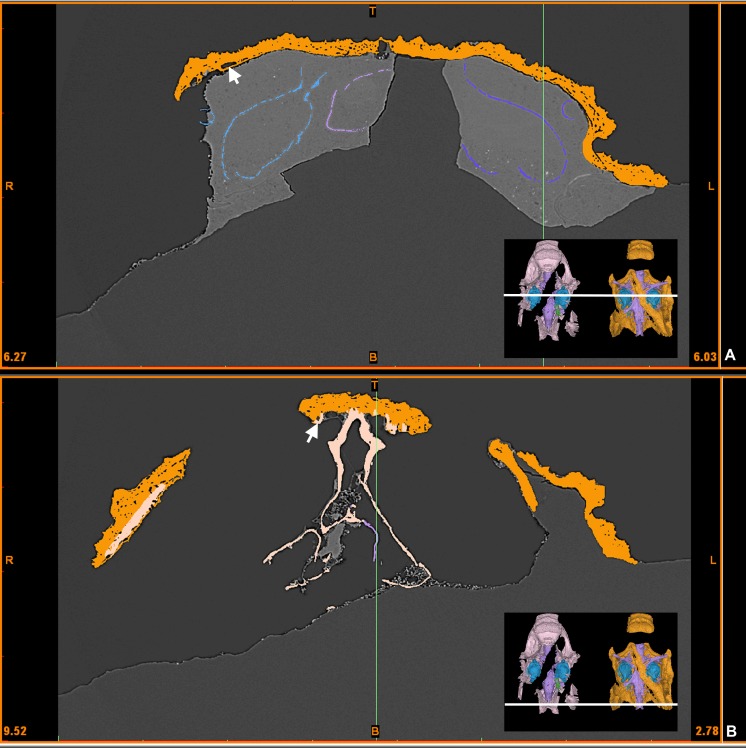
**Virtual X-ray slide in the otic (A) and occipital (B) areas of *Romundina stellina* [7], specimen MNHN.F.CPW1.** The lace pattern observed in the internal perichondral bone structures is not related to the distance to the dermal bone. White arrows indicate vascular canals at the boundary between dermal and perichondral bone layers.

**Fig 16:**
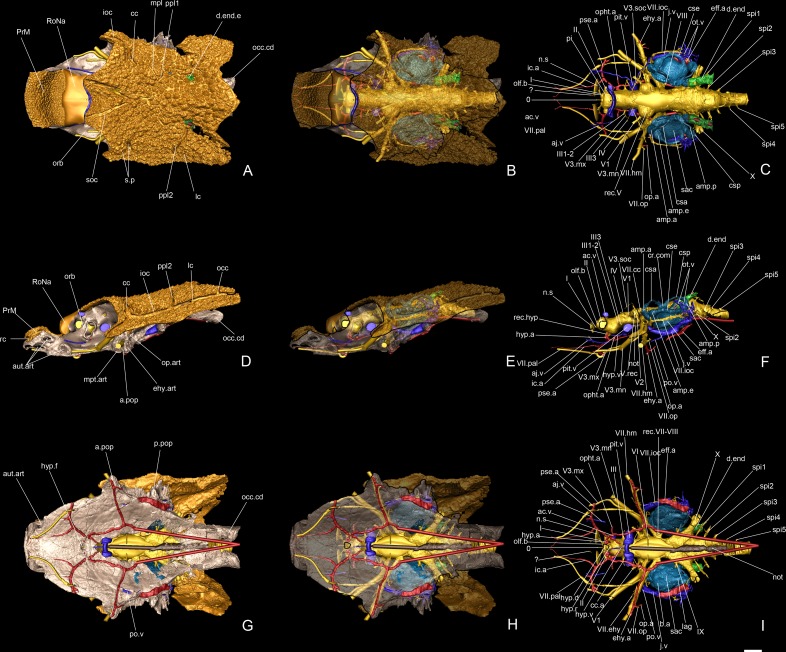
Reconstruction of the cranial anatomy of *Romundina stellina* [7], specimen MNHN.F.CPW1. Reconstruction based on Mimics (actual data, rough surfaces) and virtual prosthetics generated in Maya Autodesk (smooth surfaces, geometric shapes), in dorsal (A-C), left lateral (D-F), and ventral view (G-I), with dermal (orange) and external perichondral (pink) bones opaque (A, D, G), semi-transparent (B, E, H) and fully transparent (C, F, I). The damaged posterolateral walls and the floor of the braincase are not reconstructed. The lateral views are positioned so that the external semicircular canal is horizontal. The rostronasal capsule was reconstructed from diverse original data from [6, 7]. Scale bars are 2 mm in length.

This specimen, a three-dimensionally preserved skull, was originally enclosed in limestone matrix. Preparation with 8% formic acid buffered with tricalcium phosphate after mounting the specimen on a resin block has removed the external matrix, but most of the matrix fill inside the skull remained intact. This has preserved the delicate internal perichondral ossifications, which would otherwise have collapsed. The specimen is damaged by a large oblique crack that has resulted in the destruction of some internal structures, although the bilateral symmetry means that only a small part of the anatomy is completely lost. The posterior half of the perichondral braincase floor is much damaged and cannot be reconstructed entirely, creating some uncertainty about the pattern of arterial grooves in this region. Furthermore, the nasal capsule, which was separated from the orbital region by a complete optic fissure (a common condition in placoderms; [11]), is missing with the result that the anteriormost part of the cranial cavity is lost. Apart from these defects the specimen is almost perfectly preserved, although a small amount of vertical compression has caused some fracturing of the perichondral ossifications. The specimen comes from the same locality as the type specimen of *Romundina stellina* described by Ørvig ([7]; locality 10 in Prince of Wales Island, described in [12]; Figs 1C and 4; Lochkovian—Early Devonian). The external morphology fits exactly the description provided by Ørvig [7].

Other specimens: MNHN.F.CPW6 (*R*. *stellina*; S1 Fig) and MNHN.F.CPW2a-b (*Romundina* sp.; S2 Fig) come from the same locality.

All specimens are publicly deposited in the Collections of Paleontology of the Muséum national d'Histoire naturelle, Paris, France.

All necessary permits were obtained for the described study, which complied with all relevant regulations (Mission to Prince of Wales Island (Canadian Arctic). Mission funded by UNESCO IGCP 328, MNHN Paris and Institute of Northern Studies: Polar Continental Shelf Project N° 606–95).

### Anatomical abbreviations

See S1 Table

### Institutional abbreviations

CPW: Prince of Wales Island collection housed at MNHN, Paris, France; ESRF: European Synchrotron Radiation Facility, Grenoble, France; MNHN: Muséum national d’Histoire naturelle, Paris, France.

### Data acquisition, treatment and rendering

The sample was scanned on beamline ID19 of ESRF. The data set and acquisition protocols are related to previously published work [13].

*Mimics* v.12.3, v.13.1 and 14.0 (*Materialise*) were used for the 3D modelling (segmentation and 3D object rendering). The vascularization of the dermal bone was modelled using VGStudioMax v. 2.0 (Volume Graphics). Animations were rendered with Maya Autodesk 2011/2012 (Autodesk). Spaces such as the cranial cavity, vascular canals and nerve canals were modelled 'in positive' by filling the lumen and rendering it as a solid object.

The colour coding of the models follows, with minor modifications, the conventions established by Stensiö in his seminal papers on placoderm skull anatomy [14, 15]: dermal bones in orange; external perichondral bone in pale pink; perichondral bone of cranial cavity in lilac; perichondral bone of inner ear in light blue; endolymphatic duct in dark green; cartilage in light grey; fill of cranial cavity, nerve tracts and nerve grooves in yellow; fill of sensory line nerve twigs in light green; fill of arteries in dark red; fill of veins in dark blue; dermal bone vascularization in crimson red or pink in Fig 3; fill of notochordal cavity in brown; ventral wall of the notochordal canal in brown.

Each mask in Mimics was cut and exported into different STLs corresponding to the different anatomical structures mentioned in the text. The STL files were then treated and hierarchized by anatomical category in 3Matic (Materialise) in order to produce a 3D model in pdf format. The big size of the original STL files required reduction of the number of triangles in 3Matic, without modifying the shape of the object, in order to produce a pdf of reasonable size. 3D pdfs are available at http://paleo.esrf.eu.

## Results: Description

The present article mainly focuses on the description of anatomical structures, which is organized as the relationships between the dermal and external perichondral ossifications (Figs 2 and 3), the internal perichondral ossification wrapping the endocranial cavity (Figs 4 and 5) and the reconstructed infilled associated nervous system (Figs 6–8), the inner ears (Fig 9), the vascular system (Figs 10–13) and the parasphenoid (Fig 14). A description of the lace pattern observed in the walls of the endocranial cavity is also presented (Fig 15). A holistic reconstruction of the aforementioned structures concludes the article (Fig 16). A series of 3D pdf files allow the reader to manipulate at will the hierarchized aforementioned structures in the models of either the specimen or its reconstruction in which the missing and collapsed structures were restored.

### Skull roof

Like other placoderms, *Romundina* has a skull and shoulder girdle covered in macromeric dermal bones, externally ornamented with a dense scatter of stellate semidentine tubercles [7]. In the present specimen this covering is represented by the skull roof and premedian plate (Fig 1E). The boundaries between the component plates of the skull roof are not visible in external view, suggesting that this is an adult or near-adult individual.

The skull roof is composed of two layers of bone, which are of different origin. Dermal bone has an intramembranous mode of ossification and is not preformed in cartilage. The perichondral bone is a subtype of chondral bone; it originates from the ossification of the connective tissue surrounding a cartilaginous structure, but not the cartilage itself [16]. Recent work shows that dermal and endoskeletal bones do not have consistent embryonic tissue origins throughout the different vertebrate clades (see [17] for review).

The dermal bone is the most external layer. It is relatively thick, ornamented and densely vascularised. Under the dermal bone lies a thin and uniform compact bone (Fig 3), which we interpret as a bone formed by perichondral ossification deposited where the cartilaginous braincase touched the dermal skull roof [13]. This interpretation is supported by the fact that a number of large blood vessel canals circulate freely between this thin bone layer and the upper dermal bones. These large canals communicate both with the complex vascularisation of the dermal bone and the more scattered vasculature of the perichondral bone (white arrows in Figs 3A–3C and 15A; Fig 1 in [13]). We infer that these vessels originally were positioned between the cartilaginous braincase and dermal skull roof but were later "immured" by perichondral ossification fused onto the internal face of the dermal bone.

The vascularization of the dermal skull roof bones, including the main body of the premedian plate (Fig 13A–13C), forms a complex, multilayered honeycomb mesh radiating from the growth centre of each plate (asterisk, Fig 3C). This radiating pattern reveals the position of the otherwise non-visible external morphology of the plate sutures. In some places, notably the centre of the nuchal plate (asterisk, Fig 3C), the vascular mesh is not clearly visible. However, this appears to be due to digital segmentation problems, caused by low contrast between bone and canal-filling matrix, rather than a genuine anatomical absence. In the thin posterior part of the premedian plate, a different vascular pattern—a single layer of orthogonal, non-radiating mesh—is present (Fig 13A–13C; S2 Video; [13]).

On the external surface of the skull roof, the grooves for the sensory canals and pit lines are clearly visible (Fig 2A1). Additionally, two pairs of sensory pits are visible (s.p, Fig 2A1 and 2F; see also cu.so in [7]:figs 1A, 2A, pl.2 Fig 1). Although the median sensory pit opens externally in the central sensory line groove, they are connected to different canals of distinct nerve branches (see below). The lateral sensory pit is situated anterior to the junction between the infraorbital and central sensory line grooves (s.p, Fig 2A1 and 2F). It is noteworthy that Ørvig identified only one pair (in an intermediate position), and was unsure of its sensory nature [7].

### Braincase

*Romundina*, like all placoderms, lacks endochondral ossification. The external and internal surfaces of the braincase were in life covered by a layer of perichondral bone, which is well preserved in the specimen and documents the braincase morphology. Intervening spaces that geometrically correspond to solid braincase are now filled with sediment but were presumably occupied by uncalcified cartilage in life. As noted above, the external perichondral bone is locally fused to the basal layer of the dermal bone where the two are in contact. The imprints for the anterior and posterior semi-circular canals, for the supraorbital, infraorboital, central and main lateral sensory lines, and for the median, first and second posterior pitlines are visible on the internal side of the skull roof (respectively csa.p, csp.p, g.cc, g.soc, g.ioc. g.lc, g.mpl, g.ppl1, g.ppl2, Fig 3A–3C).

The external perichondral bone of the braincase is smooth, continuous, and in most areas avascular. However, internal vascularisation is developed in the contact surface for the premedian plate and in some areas of thick bone, notably below the nasal capsule contact margin (Fig 13). This local vascularisation cannot be associated with remodelling because of the lack of osteons as observed in the dermal bony plates (small canals with a whiter rim indicating a secondary denser bone; S1 Video; [18]). Under the posterior part of the premedian plate, the external perichondral bone is pierced by small pores, which may correspond to the lace-like organisation observed in the internal perichondral bone (see below) (see also [7]:pl.1 fig 4, pl.3 fig 5; [13]).

The internal perichondral bone, which lines the cranial cavity, inner ear cavities and associated neural and vascular canals, is much thinner than the external perichondral bone of the lateral walls of the braincase and somewhat thinner than the external perichondral bone of the braincase floor (Figs 4 and 5). The internal perichondral bone is avascular, but in a region that extends between the roof of the cerebellum posteriorly and the exit of the trochlear nerve (cranial nerve IV) anteriorly, and that also incorporates the inner ear cavities, it is perforated by numerous small holes that create a lace-like pattern (Figs 4B, 4C and 5). This condition, which has not previously been observed in any vertebrate, is clearly natural as the lace-like areas are distributed symmetrically (Figs 4B, 4C and 5). The functional significance of this pattern is obscure, but it suggests that the perichondrium (i.e. the connective tissue containing the cells at the origin of the perichondral bone production) in these areas may have contained a network of osteoblasts, interspersed with islands or patches of non-bone-producing cells such as prechondroblasts.

The infilling of the braincase reveals the presence of a pair of posteriorly oriented pointed processes below the paranuchal plates. We are unsure whether these were filled with cartilage or other tissues.

### Neurocranium and visceral arches

The neurocranium or braincase of *Romundina* is kite-shaped in outline, widening anteriorly from a narrow occiput to a widest point at the exit of the hyomandibular branch of the facial nerve (cranial nerve VII) and then narrowing again towards the snout (Fig 2). It is relatively shallow with a flat, gently concave ventral surface. *Romundina* lacks the dorsally positioned and laterally directed antorbital, ectethmoid and supraorbital endocranial processes that characterise the braincases of arthrodire placoderms ([7]:63).

Compared to a living gnathostome the most distinctive features are the broad suborbital shelves that form a floor for the eye sockets, and the position of the (missing) rostronasal capsules on the dorsal surface of the ethmoid, posterior to the premedian plate and thus well behind the anterior margin of the braincase (Fig 2). The position of the nasal capsules (digitally reconstructed RoNa, Fig 16) reflects a brain morphology radically different from that of any living gnathostome but in important respects similar to that of the galeaspid (fossil jawless vertebrate) *Shuyu* [6, 19]. The phylogenetic and evolutionary significance of these similarities is discussed below and in ref. [6].

Apart from the fissure separating the rostronasal capsule, the neurocranium of *Romundina* (and placoderms generally) is a single block without fissures or separate ossification centres. Classically it has been divided into four regions: the premedian-ethmoid, the orbital, the otic and the occipital regions (Fig 2A3 and 2A4). Because placoderms are positioned within an extant phylogenetic bracket [1–3, 6, 20] formed by living gnathostomes and lampreys (Fig 1), which are known to share highly conserved patterns of mesodermal proliferation and ectomesenchymal migration during development [21], it is useful to consider the regions in relation to these patterns. The occipital region, showing indication of segmentation in the form of a series of spino-occipital nerves, represents the anterior end of the somitic mesodermal domain. The otic region comprises the otic capsule and the anterior part of the parachordal cartilages, both formed from unsegmented head mesoderm; the possibility that an ectomesenchymal contribution from the hyoid arch neural crest stream may also be present on the lateral wall of the otic capsule is discussed below. The premedian-ethmoid and orbital regions together correspond to the part of the head created jointly by the supraoptic and infraoptic branches of the trigeminal neural crest stream in lampreys and gnathostomes [21].

As in other gnathostomes, a mandibular arch, a hyoid arch and a number of branchial arches were associated with the neurocranium of *Romundina*. Apart from the palatoquadrate, which was described by Ørvig [7], these arches are entirely unknown. However, some of their articulation facets are prominent features of the braincase wall.

### Premedian-ethmoid region

This region extends backwards from the tip of the snout and is limited posterolaterally by the anterior margin of the orbit and posteromedially by the attachment rim for the nasal capsule. This rim carries a groove representing the posterior half of the optic canal through which passed the optic nerve (g.II, Fig 2C–2E). The anterior face of the premedian-ethmoid region carries, on the dermal premedian plate, a transverse groove pierced by minute foramina: the ethmoid commissure of the sensory line system (rc, Fig 2C and 2D). This groove is continued on the suborbital and postsuborbital plates (see [7]:figs 1A, 2A).

The lateral face of the premedian-ethmoid region shows three articulation points for the autopalatine part of the palatoquadrate (aut.art, Fig 2B–2F), all lacking central perichondral ossification. Anterior and ventral to these articulations is a notch in the perichondral floor of the snout (n, Fig 2B, 2D and 2E), which probably accommodated the anterior process of the inner side of the suborbital plate of the autopalatine (see [7]:pl. 2 fig 5).

The premedian-ethmoid region does not contain any part of the endocranial cavity or major nerve tracts. However, a pair of narrow canals runs anterodorsally from the floor of the region to emerge onto the dorsal surface just posterior to the premedian plate (Fig 6A("?") and 6D; see also [13]:fig 1). These canals probably housed nerves, perhaps providing sensory innervation for the region around the nostrils. The ventral face of the premedian-ethmoid region exhibits paired grooves for the anteriormost portion of the internal carotid artery (g.ic.a, Fig 2B). The course of this artery is alternately ventral to and within the perichondral bone (ic.a, Figs 10 and 11). Anteriorly, each canal divides in the perichondral bone into two branches exiting on the anterior face of the upper lip (that is below the ethmoid commissure. A faint longitudinal midline ridge separates two shallow depressions that may represent the attachment areas of the anterior supragnathal plates (dermal palatal bones; ASG.art. Fig 2B). A shallow transverse depression delimits the upper lip posteriorly.

The upper lip is well developed in *Romundina* (see discussion and [22]). It consists of the premedian-ethmoid part of the neurocranium, including the premedian plate but excluding the rostronasal capsule, and the lateral part and rim of the orbital area (for estimated extension of the upper lip corresponding to premandibular infraoptic ectomesenchyme, see pink areas in [22]:figs 3–4).

### Orbital region

This region adjoins the premedian-ethmoid region posteriorly and extends backwards to the posterior margin of the orbits. Laterally we trace its posterior boundary as running just anterior to the foramen for the hyomandibular branch of the facial nerve (f.VII.hm, Fig 2B, 2C and 2F); this places it at the junction of the mandibular and hyoid domains of the head. Ventrally the boundary runs transversely across the braincase floor at the level of the pituitary vein. The left side of this region is best preserved. It is morphologically complex, containing a number of vascular canals, nerve foramina and attachments for extrinsic eye muscles, in addition to the eye stalk, buccohypophyseal foramen and metapterygoid articulation of the palatoquadrate. A discussion related to the nomenclature of the myodomes in the orbital area used in our article is provided in S1 Text. Internally this region contains the anterior end of the endocranial cavity, which is described separately below.

The lateral margin of the orbital region is formed by the suborbital shelf. Its dorsal face is traversed by two grooves, both positioned lateral to the anteroventral myodome (av.myo, Fig 2D) and oriented anteroposteriorly. The medial-most one is interpreted as housing the anterior branch of the jugular vein (g.aj.v, Fig 2D), which is believed to have drained the blood from the premedian plate (according to the visible connection; aj.v, Fig 13A–13C). The lateral-most one is interpreted as the palatine ramus of the facial nerve (f.VII.pal, Fig 2D), *contra* Ørvig’s opinion that it housed the anterior branch of the jugular vein ([7]:pl. 1 figs 1, 3), because it crosses the perichondral bone to continue its course anteriorly on the ventral side of the neurocranium (Fig 10A2 and 10B2), a trajectory that seems more logical for the palatine ramus of the facial nerve than for the jugular vein.

The perichondral bony cover of the eyestalk is shaped as a traditional keyhole (e.s, Fig 2C–2E), with an oblique long axis, and with its dorsal lobe slightly bigger than the ventral one. It is surrounded immediately by the dorsal myodome and an anteroventral one (the latter being situated just dorsal to the groove for the anterior branch of the jugular vein; d.myo, av.myo, Fig 2D–2E; ventral myodome of [23]). The posterior myodome lies behind the exit of the pituitary vein canal and is very deep (p.myo, Fig 2D–2E). Ventrally and slightly posteriorly to the posterior myodome is a very shallow posteroventral myodome (“scar” of [23]; pv.myo, Fig 2D–2E).

A shallow depression just below the skull roof and above the foramen for nerve IV is identified as the dorsal myodome for the trochlear nerve (d.myo.IV, Fig 2E and 2F) as suggested by Young [23].

The bottom of the dorsal myodome situated just above the eyestalk is pierced by two minute foramina for the first and second branch of the common oculomotor muscle nerve (f.III1-2, Fig 2D).

We do not see any groove just posteriorly to the eyestalk for a branch of the common oculomotor nerve leading to the anteroventral myodome, as suggested by Goujet et Young [24] and Young [23]; we believe what they identified as a groove is the posterior rim of the eyestalk.

The posterodorsal wall of the anteroventral myodome is pierced by a tiny foramen (partly hidden by the ventral rim of the eyestalk), connected to a canal leading to the ventral side of the neurocranium where it joins the internal carotid artery. This foramen probably represents the exit of the ophtalmica magna artery (f.opht.a, Fig 2D) [23].

The groove for the posterior margin of the optic nerve (g.II, Fig 2C–2E) is positioned anterodorsal to the eyestalk. Posterior to the eyestalk lies the large opening of the pituitary vein canal (f.pit.v, Fig 2D–2E). Although incomplete in our specimen, other specimens (CPW6, S1 Fig; CPW13 [6]:ext.data.fig 4) reveal that the pituitary canals of each orbit meet below the endocranial cavity. Because of its size, Goujet and Young have considered it more as an orbital sinus than a unique canal for the pituitary vein [23, 24].The hypophyseal vein canal is most likely to drain into the pituitary canal (see *Brindabellaspis* in [25]). Given its diameter, this canal may have housed other structures alongside the pituitary vein, such as an oculomotor muscle, as suggested by Stensiö [11]. However, in certain other placoderms (actinolepid and phlyctaeniid arthrodires) the corresponding canal appears too narrow to enclose both a vein and a muscle [26, 27]. This opening has previously been erroneously considered as the foramen for the trigeminal nerve by Ørvig (V, pl. 2, Fig 1). The real foramen for the first branch of this nerve is more dorsal (f.V1, Fig 2C–2E) (original correction by Goujet and Young [24]).The foramen for the trochlear nerve lies anterodorsal to it (f.IV, Fig 2D–2E). Anterodorsal to the pituitary canal exit, on the ridge separating the dorsal myodome and the pituitary vein foramen, is a minute foramen connected to the posterior branch of the common oculomotor nerve (f.III3, Fig 2D–2E).

The bottom of the posterior myodome is pierced by a minute foramen (visible only on the left side, and assuming that this is not a preservation artefact; f.VI, Fig 2E). Unfortunately, there is no canal preserved that could help for the identification of the nervous exit. However, considering the surrounding structures and the posteroventral position of the foramen, this could correspond to an exit either for a very posterior branch of the trigeminal nerve, a secondary branch of the abducens nerve, or an anterior branch of the facial nerve (although we regard this last hypothesis as improbable). The floor of the posterior myodome is pierced by the foramen of a canal leading to the internal carotid artery. This canal probably housed the orbital artery (f.orb.a, Fig 2D).

Just above the posterior myodome, two foramina in small depressions are visible; these foramina correspond to the exits of the second and third branches of the trigeminal nerve (f.V2, f.V3, Fig 2E). Immediately posterior to the third trigeminal branch foramen, the opening for a long transverse canal for the facial nerve is visible (f.VII, Fig 2C and 2E). Young ([23]:fig 2d) and Goujet and Young [24] considered this foramen as the exit for another branch of the abducens nerve in *Romundina*. The posterior wall of the orbit exhibits the large foramen for the jugular vein (f.j.v, Fig 2E).

The floor of the orbital area of the neurocranium shows several grooves and a central depression containing a hole, the hypophysial fenestra (hyp.f, Fig 2A2, 2A4 and 2B), which opens into the hypophysial recess. The infilling of the hypophysial fenestra is described below. Lateral to this hypophysial fenestra, the groove for the internal carotid artery (g.ic.a, Fig 2B) gives off three branches within a short distance. The most anterior of these is the short canal for the hypophysial artery (hyp.a, Fig 10B2), which runs medially to enter the hypophysial recess (rec.hyp, Fig 6B). A narrower canal that runs dorsolaterally to emerge on the dorsal surface of the suborbital shelf is interpreted as housing the ophthalmic artery (opht.a, Fig 10B2). Posterior to the hypophysial artery, the third branch is represented by a groove that runs posteromesially but, unfortunately, ends at the broken edge of the braincase floor before reaching its destination. For this reason, and also because it is unclear whether the vessel in this groove actually connected with the internal carotid artery (there is a small discontinuity between them), we are not able to identify it with confidence. The internal carotid continues its course anteriorly from the junction with the hypophysial artery before giving off a laterally directed pseudobranchial artery (g.pse.a, Fig 2B; pse.a, Fig 10A2 and 10B2). Posterior to the ophthalmic artery, the course of the internal carotid artery can be traced back to the anterior part of the otic region.

Because of the damaged condition of the braincase floor we are unable to determine whether the pituitary vein was exposed ventrally in the midline. However, other acanthothoracid specimens from the Lower Devonian of Saudi Arabia show that the pituitary vein canal does not open on the ventral side of the neurocranium [28]. Lastly, a short unpaired canal, slightly shifted to the right from the midline, has been interpreted as an hypophyseal vein by Ørvig ([7]; pl. 1 Fig 3; hyp.v, Fig 2B). We find that the anterior part of this vessel was internal and connected to the hypophyseal recess. Its identity is uncertain, but it may represent an unpaired median hypophyseal vein (?c.hyp.v, Fig 5).

### Otic region

The otic region extends between the anterior postorbital process anteriorly and the vagus nerve exit (Figs 2A3, 2A4 and 4) posteriorly. The anterior postorbital process is pierced by the hyomandibular branch of the facial nerve; f.VII.hm, Fig 2B, 2C and 2F). The anterior side of the distal extremity of the anterior postorbital process shows an unossified area surrounded by a rough-textured protruding ring (like those in the pre-ethmoid area for the autopalatine attachment), with a collapsed mediodorsal wall. This articulation area is most likely related to the attachment of the metapterygoid part of the autopalatine (mpt.art, Fig 2F). Immediately posterior to the exit of the hyomandibular branch of the facial nerve lie two further articulation areas of similar character, one located above the other. We interpret these areas as hyoid arch articulations, the upper one for the opercular cartilage and the lower one for the epihyal (= hyomandibula; see Trinajstic et al [29] for discussion) (op.art, ehy.art, Fig 2F), or possibly as articulations for a single bifid hyoid arch element (possibly acquired by convergence with sarcopterygians). This disposition of articular surfaces allows the position of the mandibular/hyoid arch boundary (and thus, by inference, the boundary between the trigeminal and hyoid neural crest streams) to be mapped very precisely to the tip of the anterior postorbital process.

Between the anterior and posterior postorbital processes on the lateral side of the neurocranium, just below the edge of the skull roof, lies a large longitudinal groove (ehy.myo, Fig 2F). This groove is devoid of any foramen, except in its most posterior extremity (where it connects to the glossopharyngeal nerve). The right antimere of this groove shows four shallow rounded depressions medially. We interpret this groove as a muscle attachment area, most probably housing hyoid arch and possibly branchial arch musculature. It may correspond to the postarticular pit described in *Brindabellaspis* by Young ([25]:26, text-fig 8, part.p). Just posterior to the attachment areas for the opercular and epihyal elements (that is, on the anterior wall of the previously described groove), a foramen connects to a posteriorly oriented sub-branch of the hyomandibular branch of the facial nerve. We interpret this as the epihyal branch (f.VII.ehy, Fig 2). The ventrolateral edge of the otic area exposes two consecutive depressions (?ba1.art,? ba2.art, Fig 2B and 2F) that are considered as the attachment areas for the first two branchial arches. The first one is situated below the posterior end of the muscle attachment area; the second one is located below the groove for the jugular vein. This disposition would thus be more anterior than the posterior postorbital process on which the five branchial arches are supposedly attached [26], and would imply that the branchial basket has multiple articulations to the neurocranium at the level of and anteriorly to the posterior postorbital process. This is somewhat reminiscent of Stensiö's opinion in which the branchial basket of placoderms was elongated according to his elasmobranch model (although located behind the neurocranium in extant chondrichthyans; [11, 15]). Heintz, using the example of *Dinichthys*, refuted the idea of a branchial apparatus positioned behind the head for placoderms (i.e. within the thoracic armour), and proposed with great caution and without any physical evidence that the branchial basket would be located ventrally and in the posterior part of the head, as in teleostomes ([30]:199–202, text-figs 88–89). It is noteworthy that the submarginal ("opercular") plate of such big predators was unknown until Carr's reanalysis of *Heintzosteus* [31], in which the opercular function may not have been fulfilled; it was thus likely that the branchial baskets and openings were located ventrally under the head, in the space between the postsuborbital plate of the cheek cover, and the reflected lamina of the interolateral and anterior lateral plates of the thoracic armour. In flattened forms such as the rhenanids *Gemuendina* or *Jagorina*, the branchial openings are dorsal; in other flattened species such as the petalichthyid *Lunaspis*, three paired elements of the branchial apparatus are visible ventrally under the occipital part of the head, but there is no certainty regarding the branchial opening [32]. In derived arthrodires such as *Pholidosteus* (see [33]:fig. 2), the submarginal plate is fused to the rest of the skull roof and cheek cover and cannot perform an opercular function.

The ventral side of this neurocranial region is crossed by the internal carotid artery grooves, which give off lateral branches for the epihyal arteries (g.ic.a, g.ehy.a, Fig 2B). The epihyal artery branches off at a level immediately ventral to the canal for the hyomandibular branch of the facial nerve (Fig 2B and 12B–12E), and parallels that canal to its exit at the tip of the anterior postorbital process. Posteriorly, the lateral margin of the floor of the otic region carries a groove interpreted as housing the laterodorsal artery (g.ld.a, Fig 2B). The lateral wall of the region contains a wide jugular vein canal. This canal opens anteriorly in the posterior wall of the orbit, runs internal to the abovementioned muscle attachment area in the braincase wall, and continues as an open groove posteriorly (g.j.v, Fig 2F). Two vertical branches arise from the posterior part of the jugular canal and trace a curving course around the inner ear to connect with the vasculature of the dermal skull roof; they are interpreted as carrying the otic veins (ot.v, (Fig 13D and 13E).

### Occipital region

This region extends posteriorly from the canal for the vagus nerve (c.X, X, Fig 4B and 4C) to the occipital condyle (occ.cd, Figs 2B, 3D and 3E). This region is unfortunately rather badly preserved. It narrows gradually towards the occipital condyles and its side walls slope inwards dorsally such that the floor of the occipital region is its widest part. Posteriorly the floor is split by a notochordal fissure, which is continued anteriorly by an enclosed notochordal canal that reaches as far as the oblique crack that runs through the specimen (ch.c, Fig 4D). The exits for the different branches of the vagus nerve are not visible, because the lateral walls of the braincase are not preserved in that area (although the course of the vagus nerve is shown by the perichondral bone lining of its canal). Further posteriorly, four foramina on the left side and two on the right side correspond to the exits for the spino-occipital nerves 2–5 and 1 and 5 respectively (f.spi2, f.spi3, f.spi4, f.spi5, Fig 3D–3E). The occipital condyles also consist of perichondral bone, although it is porous and thicker than the layer wrapping the neurocranium, the endocranial cavity and the inner ears. Its internal organization displays a random pattern different from that encountered in the dermal bone.

### Endocranial cavity

The endocranial cavity is not preserved anterior to the ethmoid fissure (i.e. anterior to the optic nerve), because the rostronasal capsule (which closes the brain cavity anteriorly) is lacking in specimen MNHN.F.CPW1. In the models obtained by means of from Mimics, the endocranial cavity was finished off anteriorly with a featureless rounded end (Figs 6–8) However, the rostronasal capsule has been described by Ørvig [7] (pl. 5, figs 2–5) from a detached specimen: it houses two small cavities for the olfactory lobes posteriorly, connected by numerous small foramina for the fila olfactoria to the nasal cavities, which open anteriorly. The olfactory lobes and tracts are thus both extremely short. These features were recreated on Maya Autodesk using geometric volume primitives, which generate structures with smooth surfaces that can easily be distinguished as "prosthetics" (Fig 16; see also [6]:figs 2c-f,3).

In dorsal view, the endocranial cavity is tubular, with three more or less pronounced bulging structures at the levels of the optic, trigeminal and vagus nerves. It is narrowest just posterior to the acoustic nerve (c.VIII, Fig 4B and 4C), from where it widens slightly until the vagus nerve (c.X, Figs 4B, 4C and 5) before narrowing again posteriorly. In lateral view, the part of the cavity that lies posterior to the trigeminal recess is also essentially tubular, with a maximal depth just posterior to the vagus nerve at the point where a pair of (most probably vascular) canals leaves the cavity posterodorsally, before turning ventrally and reentering the endocranial cavity (c.vasc, Fig 5). However, anterior to the trigeminal recess, the endocranial cavity has a distinctive profile in lateral view. The region between the trigeminal recess and hypophysis (approximately corresponding to the mesencephalon of the brain) is arched, with both floor and roof rising quite strongly in the middle. The hypophysial recess is directed anteroventrally, and at the point where this recess joins the endocranial cavity, the latter bends dorsally by almost 90 degrees to extend anterodorsally up to the optic foramen and the optic fissure. There is no visible pineal recess, but considering other specimens, we would expect it to be low and located in the rostronasal capsule (see [6]:extended data fig 4).

As already stated above, the perichondral bone layer wrapping the endocranial cavity and the inner ears is not uniform, but rather presents a “lace” pattern between the trigeminal and vagus nerves (Figs 4 and 5). The maximum number and density of perforations occurs between the acoustic and vagus nerves.

### Central nervous system

The endocranial cavity has been digitally filled in order to make it easier to visualize. The medial oblong part obviously contained the brain, meninges and cerebrospinal liquid. Since the brain is not preserved *per se*, a complete filling of the cavity has been performed, but it should be noted that the brain rarely fills the cavity completely in fishes and can in some cases be much smaller [34–36]. The general shape of the endocranial cavity has been described above. We will here focus on the canals for the lateral nerves.

The optic nerve tract (II, Figs 6 and 7) has been reconstructed by considering the curvature of its preserved posterior wall: lacking the rhinocapsular bone, we cannot be sure of its exact diameter. It appears as the largest of all the cranial nerves but may in fact have been somewhat flattened anteroposteriorly. The oculomotor nerve (III, Figs 6 and 7) exits the middle of the lateral wall of the cranial cavity and divides into two branches, both directed into the orbital wall; only the ventral one is completely preserved, but given the orientation of the dorsal branches and the foramina on the orbital wall, there is no doubt as to their respective homologies and exits. The dorsal branch divides into two rami that both innervate the superior myodome (III1-2, Fig 6A), while the ventral branch exits between the eyestalk and the pituitary vein foramen (III3, Fig 6A). The trochlear nerve tract (IV, Fig 6A) is not entirely preserved and we can only assume its homology with the direction of the bud originating from the endocranial cavity and the corresponding foramen in the orbital wall (f.IV, Fig 2E).

The trigeminal recess is well developed (rec.V, Figs 6 and 7), and bulges laterally and posteroventrally into two hemispheres. Because of the oblique crack, only the left side is properly preserved. Similarly to what occurs in *Brindabellaspis* (see [25]:text-figs 11–12), but contrary to *Kujdanowiaspis* (see [11]:figs 28, 30), the different branches of the trigeminal nerve exit separately from different parts of the trigeminal recess, i.e. there is no common trigeminal nerve trunk. Four branches emerge from the recess: the most dorsal one (V.d, Fig 6A, 6C and 6D) could either be connected to the profundus sensory line system or be a vascular vessel. Just anteroventral to it, the real profundus branch (V1, Fig 6A, 6C and 6D) is directed anterolaterally and emerges in the orbital wall posterior to the eyestalk (f.V1, Fig 2C–2E). Just before the exit of the nerve in the orbit, a thin branch separates dorsally from the main trunk but does not exit in the orbit itself. Posteroventral to the V1 canal, an anterolaterally directed canal leaves the trigeminal recess and exits in the orbit just above the posterior myodome (f.V2, Fig 2E; V2, Fig 6A, 6C and 6D). This canal most probably transmitted the second branch of the trigeminal nerve, although it is also possible that it is part of the vascularization. The third branch of the trigeminal nerve canal branch is very large (V3, Fig 6A, 6C and 6D); it is about twice the size of V1 or V2. The canal sprouts from the posteroventral part of the trigeminal recess. The median part is not preserved, and we cannot be certain of the exact origin of this branch. The nerve exits on the roof of the posterior myodome, just posteriorly to the foramen for the branch V2. The faint and shallow grooves on the orbital platform indicate that this branch divides into a large mandibular ramus laterally and a thinner maxillary ramus that runs anteriorly along the lateral side of the suborbital shelf (its path is traceable until the attachment area for the palatoquadrate; g.V3.mn; g.V3.mx, Fig 2C–2F). The main trunk of V3 may have share a groove with the main trunk of the facial nerve, but its length is uncertain.

The abducens nerve is unfortunately not preserved, possibly because of the presence of the oblique crack together with the fact that its trunk is usually very thin and fragile. Its presence is nevertheless evidenced by a foramen for this nerve at the bottom of the posterior myodome (f.VI, Fig 2E). The facial and acoustic nerves share a short recessus in the ventrolateral part of the endocranial cavity (VII, VIII, rec.VII-VIII, Fig 6). The canal for the facial nerve exits in the orbit just posterior to the third branch of the trigeminal nerve. It is noteworthy that despite exiting very close to each other, the two nerves do not share the same foramen, unlike what can be seen in *Brindabellaspis* (i.e. both canals merge into one before exiting in the orbit; [25]:fig 8). The facial nerve then divides into palatal and hyomandibular branches in the orbit just mesially and in front of the foramen for the jugular vein (here also the grooves are very faint; Figs 2C–2E and 16). The palatal branch is directed anteriorly across the orbit floor, while the hyomandibular branch runs along the posterior wall of the orbit before piercing the bone of the anterior postorbital process. This hyomandibular branch protrudes two more slender branches. The first, which leaves the hyomandibular branch ventrolaterally, is most clearly visible on the left side of the specimen; its affinity is uncertain, but we interpret it as the epihyal branch of the facial nerve because its visible course follows the epihyal artery (VII.ehy, Figs 6A, 6B, 7C, 7D, 11C2 and 11D2). The second exits on the posterior side of the anterior postorbital process, on the anterior margin of the myodome groove. We consider it as an opercular branch of the facial nerve (VII.op, Figs 6, 10 and 11).

A thin dorsally oriented canal leaves the main V3 canal just distally to the junction with facial nerve canal. This canal then sprouts into several branches. The most dorsal one describes a wide anterodorsally oriented loop and connects to the supraorbital sensory line (V3.soc; Figs 6 and 7). Another branch is connected to the two sensory pits (s.p) visible on each postorbital plate; The most medial pit is itself connected to the loop leading to the supraorbital sensory line (V3.sp, Figs 6 and 7). Minute branches exit in the orbit.

Another thin branch exits the facial nerve and similarly joins the infraborbital sensory line (VII.ioc; Figs 6 and 7) before surrounding the inner ear laterally, while a ramification connects to the central sensory line dorsally (VII.cc; Figs 6 and 7).

The acoustic nerve (c.VIII, Fig 5; VIII, Fig 6) shares a short ventrolateral recess with the facial nerve, before entering the anteroventral part of the sacculus. Just before entering the inner ear, the canal divides into short anterior and posterior branches.

The glossopharyngeal nerve (c.IX, Fig 5; IX, Figs 6, 8 and 9) is bifid and divides shortly after leaving the endocranial cavity (this division is visible only on the right inner ear; Fig 9B5 and 9B6). The dorsal (posterior) branch enters the inner ear just medioventrally from the posterior ampulla and utriculus. The ventral (anterior) branch is incomplete, but its continuation can be identified as a canal below the internal foramen for the endolymphatic duct that can be seen on the medial side of the right inner ear. It is noteworthy that in other taxa, the glossopharyngeal nerve runs below or at the very base of the inner ear. The nerve exits the inner ear laterally through a canal situated just above that for the jugular vein (jv, IX, Fig 9A4, 9A5, 9B3 and 9B6). It then connects dorsally in a complex way with the jugular vein canal, from which two vertical canals issue dorsally and then curve mesially around the inner ear cavity. These two canals merge just before entering the skull roof vasculature. It seems that the main canal is directed towards the median pit line. It is however difficult to determine which canal corresponds to blood or nerve path (or both; ot.v, Figs 10B1, 11 and 16). We identify it hesitantly as the otic vein.

The exit of the vagus nerve (c.X, Fig 5; X, Fig 6) is not preserved, but the nerve tract is well preserved and quite complex. From the vagal recess two branches exit: a small dorsal and a much larger ventral one, the latter trifurcating into two anterior branches (one large dorsal and a more slender ventral one) and one posterior branch. Additionally, two thin ventral branches emerge from the vagus trunk; the proximal one is directed ventrolaterally (X0) and joins the more distal one which is directed anterolaterally and surrounds the posterior utriculus and lagena of the inner ear (X1, Fig 8). This curved thin branch can be interpreted in two ways: 1) it is the anteriormost branch of the vagus nerve, and is related with the branchial area (see *X1*, in [15]:fig 66); 2) it is associated with the otic lateral sensory line system of nerve IX (see *rlc*, in [15]:fig 66). Because of the ventral disposition of this branch, we favour the former hypothesis. As for X0, Stensiö identified a similar branch on the ventral side of the endocranial cavity of *Kujdanowiaspis*, but did not label nor described it (see [15]:fig.44).

The vagus nerve of *Romundina stellina* thus has five branches (X0-4, Fig 8B), *contra* seven for *Kujdanowiaspis* (including the “r.lc” and “n.l.l” related to the sensory line system; [15]:fig 66), while Young ([25]:35–36) recognizes in *Brindabellaspis* only three ramifications of the main vagal canal, plus a number of dorsal branches innervating the posterior pitline.

It is noteworthy that the five vagal branches in *Romundina* separate at about the same level from each other. By contrast, Stensiö ([11, 15]:fig.66) illustrates in *Kujdanowiaspis* an anteroposterior succession of divisions. *Macropetalichthys* illustrates an intermediate disposition ([15]:fig 60).

A last vertical branch not connected to any larger structure is visible posteriorly to the vagus recess and dorsally to the first craniospinal nerve (X.d, Fig 8). Its base shows an extremely short bifurcation anteriorly, possibly indicating a connection towards the vagal complex.

### Inner ear and endolymphatic duct

Like all placoderms, *Romundina* has a characteristic gnathostome inner ear with three semicircular canals, rather than two as in lampreys and fossil jawless vertebrates [19, 37]. The inner ears are positioned lateral to the endocranial cavity and are separated from it by a distinct gap that in life corresponded to a complete cartilage wall. This condition is characteristic for placoderms [11], and modern elasmobranchs, but in bony fishes, tetrapods and early chondrichthyans the inner ear cavity is at least partly confluent with the endocranial cavity [38].

The inner ear cavity of *Romundina stellina* appears as a compact space with relatively short semicircular canals. The layout of the semicircular canals resembles that in bony fishes and tetrapods, and lacks the specializations seen in elasmobranchs [38]. A common recess for the anterior and external utriculi (rec.ut.ae, Fig 9) is visible just anterior to the sacculus (sac, Fig 9) and ventral to the anterior and external ampullae (amp.a, amp.e, Fig 9). The posterior utriculus (ut.p, Fig 9) is extremely short, and is noticeable only because of its slight bulge distinguishing it from the posterior part of the sacculus (sac, Fig 9) and the ventral part of the posterior ampulla (amp.p, Fig 9). It is noteworthy that the utriculi are all extremely short, hardly distinguishable; in other words, there is no slender continuation of the semicircular canals, such as those illustrated for *Kujdanowiaspis* by Stensiö ([15]:fig 61A-C). The anterior and posterior semicircular canals meet at the median part of the inner ear and form a *crus commune* (cr.com, Fig 9), the generalized condition for gnathostomes other than recent elasmobranchs [38]. The sinus superior below the *crus commune* is very short (s.s, Fig 9). In dorsal view the courses of the three semicircular canals do not intersect with each other. This is again similar to the condition in tetrapods and bony fishes, whereas in chondrichthyans (both holocephalans and elasmobranchs) the posterior and external canals frequently overlap [38]. The inner ear cavity is not completely lined with perichondral bone: the anterior and posterior semicircular canals (csa, csp, Fig 9) are not preserved at mid-course, and the lateral face of the external semicircular canal (cse, Fig 9) is unossified. None of the semicircular canals appears to have contacted the skull roof, as they have not left any visible impressions on its inner face.

The canal for the facial nerve (VII, Fig 9) shows an oblique course on the anteroventral wall of the sacculus. Just posteriorly to this canal and on the medial side of the inner ear, two foramina are visible for the entrance of the anterior and posterior branch of the acoustic nerve (VIII, Fig 9). Posterior to these, and just medial to the posterior ampulla (amp.p, Fig 9), one can see the innermost part of the endolymphatic duct, which is oriented posterodorsally and is slightly oblique; (d.end.i, Fig 9). The duct is longer than assumed by Ørvig ([7]:fig 1). Posteroventral to the endolymphatic duct, two foramina correspond to two branches of the glossopharyngeal nerve (IX, Fig 9). The anteroventral and posteroventral extremities of the sacculus are developed into distinct ventrally directed bulges (b.a, b.p, Fig 9), which may have housed the saccular and lagenar maculae. The angle between the anterior and posterior bulges is about 70°. Between the anterior and posterior bulges, the floor of the inner ear cavity has collapsed on both sides. The lateral wall of the sacculus reveals only two canals, situated approximately below the posterior ampulla. The most anterior one is connected to the jugular vein (j.v, Fig 9); the posterior one is attributed to a lateral expansion of the glossopharyngeal nerve (IX, Fig 9), before the latter divides itself into pharyngeal, pre- and post-trematic branches.

The inner ears of *Romundina* as a whole are bulkier than those of *Kujdanowiaspis* and of *Brindabellaspis*, the latter showing the smallest ones. However, it is noteworthy that the vagus recess is much more developed in *Brindabellaspis* than in *Romundina* or *Kujdanowiaspis* where it is of similar proportions. As well, the semicircular canals are very short in *Brindabellaspis*, and there is no crus commune (see [15, 25]).

The crus commune observed in *Romundina* is very reminiscent of the condition observed in the fossil elasmobranchs *Cladodoides* and *Pucapampella* [39, 40]. This structure is also observed in chimaeroids and osteichthyans, but also lampreys and many osteostracans [5, 38, 39, 41]. Whether this condition constitutes a primitive state of character in vertebrates or whether it was acquired independently in different lineages remains controversial.

The endolymphadic duct is a tube that links the inner ear to the external environment (d.end, Figs 7–9). Among extant vertebrates, only chondrichthyans retain an open endolymphatic duct in the adult stage. Because it pierces the skull roof, the wall of this tube in *Romundina* is composed of dermal bone dorsally and perichondral bone ventrally. As a consequence, the dorsal part of the endolymphatic duct is highly vascularised, like the rest of the skull roof. More surprising is the presence of a single very slender blood vessel ventral and parallel to the canal (arrow, Fig 9E and 9F). Unfortunately, the canal is not entirely preserved, and we cannot determine its most anterior trajectory. The endolymphatic duct of *Romundina* is directed posterodorsally and very slightly obliquely, not posterodorsolaterally as in arthrodire placoderms, and not vertically as in modern elasmobranchs.

### Vascular system

Here we describe the main vasculature (i.e. veins. and arteries) that can be inferred from preserved canals and grooves in the skull of *Romundina*. The vascularisation of the dermal bone is treated separately.

#### Arteries

Arteries are indicated in red on the model. All of them are situated on the ventral side of the neurocranium floor, except for a few short branches that reach internal organs.

The course of the laterodorsal artery itself is only preserved on a very small fraction of its anterior part (on the left side next to the midline and below the facial nerve (ld.a1, Figs 10 and 11). The connection with the efferent branchial arterial complex is not preserved. However, given the visible elements, it seems to resemble more the disposition seen in *Kujdanowiaspis* than in *Brindabellaspis*. The large diameter of the most lateral arterial element indicates that it corresponds to the main trunk of the efferent branchial artery rather than to the branch for the first branchial arch (eff.a, Figs 11 and 12). It extends anteriorly on the lateral most part of the ventral side of the neurocranium floor until the level of the middle part of the epihyal myodome that it seems to have irrigated (ehy.myo, Fig 2B). In this hypothesis, the (non preserved) efferent branches would have been connected to this common branchial trunk rather than branching separately on the anterior or posterior part of the laterodorsal artery. This condition is not known in other ganthostomes. This interpretation is supported by a specimen of *Romundina* sp., from the same locality as *R*. *stellina* (S2 Fig), which shows a single vascular groove on each side (white arrows) connecting the groove of the lateral trunk to the groove of the laterodorsal artery. Separate efferent branches for the branchial arches are not visible.

The common carotid artery (cc.a, Figs 10 and 11) connects with the laterodorsal and the epihyal arteries just below the chiasma between the third branch of the trigeminal and the facial nerves. It is noteworthy that in *Brindabellaspis*, a branch of the laterodorsal artery divides from the main trunk at the level of the vagal nerve lateral exit, runs medially and around the inner ear before splitting again into a supraorbital branch of the orbital artery, underlying the course of the facial nerve ([25]:text-fig 11, *lda*, *lda1*, *ebr*, *a*.*so*, *10a*, *7*). It is most likely that a similar pattern occurs in *Romundina stellina*.

Like in *Brindabellaspis*, the common carotid artery of *Romundina* divides into an anterior branch and an epihyal artery branch (ehy.a, Figs 10 and 11), the latter paralleling the course of the facial nerve and then its hyomandibular branch, until the tip of the ventral side of the anterior postorbital process. Just after this bifurcation, a short internal dorsal orbital arterial branch exits in the posterior myodome (orb.a, Fig 11).

The anterior branch of the common carotid artery then divides in a possible posteriorly directed branch (it is difficult to tell whether this branch is really related to the main course of the artery), an internal dorsal ophthalmic branch exiting in the ventral myodome below the eyestalk (opht.a, Figs 10 and 11), an internal hypophyseal artery (hyp.a, Figs 10 and 11) (that connects the lateral corner of the hypophyseal recess), and an anterior internal carotid artery (ic.a, Figs 10 and 11). This internal carotid artery then divides itself into an unidentified anterior branch and a lateral efferent pseudobranchial arterial branch (pse.a, Figs 10 and 11), overlapped by the palatine ramus of the facial nerve and exiting laterally on the ventral side of the neurocranium floor, just behind the most posterior attachment area for the autopalatine (aut.art, Fig 2). The anterior course of the internal carotid artery is not complete, partially enclosed within the perichondral bone, and exits anteriorly into two branches on the anteroventral tip of the snout (below the ethmoid commissure gutter; Figs 10 and 11).

#### Veins

Veins are indicated in blue on the model. The venous web is not as well exposed as the arterial web.

The anterior jugular vein (aj.v, Figs 10 and 11) shows part of its anteroposterior course on the ventral wall of the orbit, between the eyestalk medially and the palatine ramus of the facial nerve laterally.

The anterior cerebral vein (ac.v, Figs 10 and 11) is overlying the optical nerves just below the anteromedian part of the skull roof. A pair of thin pair of paramedian vertical canals link its posterior side to the endocranial cavity.

The pituitary vein (pit.v, Figs 10 and 11), if it occupied the whole pituitary canal space, is huge. It is hence more likely that the visible pituitary space was more a sinus than a simple canal. It is uncertain on this specimen if the pituitary veins of both orbits constituted a continuous structure. As well, we cannot assess if the hypophyseal vein (hyp.v, Figs 10 and 11), apparently a single and unpaired structure, connected the hypophyseal recess with the pituitary vein (see further discussion of the hypophyseal vascularization area). The study of the present specimen combined with the observation of other specimens (see [7]) reveals that the course of the hypophyseal vein and its connection with the pituitary vein are internal, although very close to the layer of perichondral bone of the palate.

The jugular vein (j.v, Figs 10 and 11) is the longest venous tractus preserved on the specimen. It extends from an opening on the posterior wall of the orbit until the level of the vagal nerve posteriorly.

A ventral branch, identified as a postorbital vein (po.v, Figs 10, 11 and 16) joins the main jugular vein internally, at the level of the anterior and external ampullae of the inner ear. Its anterior extension is unknown after a short external course on the ventral side of the neurocranium floor.

Just anteriorly to and at the level of the glossopharyngeal nerve and the posterior ampulla of the inner ear, the jugular vein collects two vertical, curved canals descending from the vasculature of the dermal skull roof (and tentatively referred to as otic veins; see above). These canals follow the inner ear curvature and are interpreted as carrying the otic veins (ot.v, Fig 13D and 13E). Considering their position and relationships with other canals (i.e. the glossopharyngeal nerve canal), it is most likely that these canals also included nerves (branch IX). Indeed, they connect to each other just before joining the vasculature of the skull roof (between the dermal and perichondral bone). From there, they connect to the median pitline.

## Discussion

### Data quality

*Romundina stellina* is, to date, the only placoderm in which the cranial anatomy has been investigated using synchrotron X-ray microtomography. Previous investigations of placoderm cranial anatomy include the landmark studies by Stensiö [10, 11, 14, 15, 37, 42–54] based on a combination of mechanical preparation and serial grinding, Young's description of the skull of *Brindabellaspis* from an acid-prepared specimen, and Goujet's description of the head of *Dicksonosteus* from needle-prepared specimens [25]. Stensiö's work is in some respects most directly comparable to the present study, because the grinding-series technique (whereby the specimen was ground down at 0.2 mm or 0.5 mm intervals and drawings of the ground surfaces used as templates for constructing a wax model of the internal spaces) can be regarded as a conceptual template for our scanning and modelling approach. Unfortunately, Stensiö's interpretations are sometimes affected by his prior expectation that placoderm anatomy should conform to both an elasmobranch model and an archetypal gnathostome pattern, and because he scarcely presents his primary data. This makes it difficult to judge how far his conclusions can be trusted (see also Stensiö's subjectivity in [55]). Indeed, in his seminal work on the anatomical studies on the arthrodiran (i.e. placoderm) head published in 1963 [11], Stensiö consistently compares structures with those of elasmobranchs. This idea reflects his classification, in which Arthrodira (i.e. Placodermi) constitute with Holocepahli the group Placodermata. He does not provide more information, accepting Ørvig's demonstration according to which extant Holocephali are supposed to be derived from Ptyctodontida (or a more primitive related group; [56]). The Placodermata and the Elasmobranchii constitute the super-class Elasmobranchiomorphii [11, 14, 15].

A preliminary study where section drawings from Stensiö's *Kujdanowiaspis* grinding series were compiled and modelled in 3D using Mimics (the same modelling software as in the present study) revealed significant differences from the published reconstruction drawings (pers. obs., VD & PEA). This is particularly problematic given that Stensiö's reconstructions, especially *Kujdanowiaspis*, are very widely cited and substantially condition our ideas of placoderm anatomy. They are also frequently used as outgroups in phylogenetic analyses of the gnathostome crown group. Additionally, the validity of the chondrichthyan model as representative of a generalized gnathostome condition is increasingly challenged by new information and publications (e.g. [3, 6, 57]).

Young's *Brindabellaspis* study is not affected by this problem, but all the anatomical information about endocranial structures had to be extracted from the views of the acid-prepared internal perichondral bone architecture that could be obtained from natural breaks in the specimen [58], and thus does not allow virtual dissection in the same way as the present model of *Romundina*. The present study thus represents the most detailed and, we believe, the most accurate visualization to date of one of these early gnathostomes.

The original scan data are deposited at http://paleo.esrf.eu where they can be accessed freely.

### *Romundina* and the origin of gnathostomes

In contrast to Stensiö's view that placoderms have chondrichthyan affinities [11, 14, 15, 54], the majority opinion in recent years has been that they are members of the gnathostome stem group [1–3, 6, 59, 60]. This suggests that they may be uniquely informative about early jawed vertebrate morphology and the jawless-to-jawed vertebrate transition. Further impetus has recently been given to this line of enquiry by the hypothesis that placoderms are not a clade but a paraphyletic array encompassing the most crownward part of the gnathostome stem lineage [1, 2]. The analyses that recover placoderm paraphyly place the group at the base of the array, i.e. as the sister groups to all other vertebrates with jaws. A number of forms including antiarchs and *Brindabellaspis* are characterized by nasal capsules located just in front of the orbits. *Romundina* also shares this attribute, and a phylogenetic analysis using the matrix of Zhu et al. [3] places it in the same region of the phylogeny as the aforementioned taxa [6] (between the first gnathostomes and the Arthrodira). The anatomy of *Romundina* is thus of great interest to investigate the origin of the jawed vertebrate condition. This is all the more so given that antiarchs had cartilaginous braincases that are known only from attachment scars on the inside of the dermal skull roof [61]. At present only *Romundina*, *Brindabellaspis* and *Jagorina* [52] show the endocranial anatomy associated with posteriorly positioned nasal capsules.

The most striking feature of the anterior endocranial cavity of *Romundina* (which is also observed in "dolichothoracid" arthrodires) is its extreme shortness: the optic nerve tract is almost at the anterior end, and the olfactory lobes and tracts are extremely short [6, 7]. Together with the anteriorly directed hypophysial recess, this creates the impression of a truncated brain sprouting two nasal sacs (dorsally) and a hypophysis (ventrally) from its anterior end—a very different layout to the conventional extant gnathostome brain with long forebrain, moderately to very long olfactory tracts, and a posteriorly positioned and ventrally oriented hypophysis (e.g. [6, 38, 62–65]). *Brindabellaspis* appears to have a very similar organization, including the anterior orientation of the hypophysial recess and the abrupt dorsal turn of the anteriormost part of the endocranial cavity [25]. This endocranial morphology bears a clear similarity to those seen in living and fossil jawless vertebrates. Lampreys and hagfishes both have short forebrains and effectively non-existent olfactory tracts, the olfactory bulbs being in direct contact with the olfactory lobes of the brain [65]. The same applies, as far as can be determined from the endocranial cavity, to the fossil group Osteostraci [37].

The recently described endocranial cavity of the galeaspid *Shuyu zhejiangensis* [19] is of particular interest in this comparative context. The clades Galeaspida and Osteostraci both belong to the paraphyletic assemblage of ostracoderms, or jawless stem gnathostomes. Galeaspida have well separated left and right nasal sacs and a hypophysial recess that opened anteriorly between the nasal sacs in the supraoral space. This suggests that their nasal sacs and hypophysis developed from three separate placodes like those of jawed vertebrates, not from a single placode as in cyclostomes (lamprey and hagfish [19, 21, 66]). The supraoral space opens ventrally through the mouth and dorsally onto the outer surface of the head, in the form of a nasohypophysial opening. Interestingly, no evidence for a bony or cartilaginous separation between the oral and nasohypophysial components of the cavity has ever been found; an apparently similar case occurs in the Heterostraci, though the unmineralized (and hence unpreserved) endoskeleton of this group limits our understanding of their anatomy [5]. The anterior endocranial cavity of *Shuyu*, which has also been imaged by synchrotron X-ray microtomography, is remarkably similar to that of *Romundina*: the arched mesencephalic region, anteriorly oriented hypophysial recess, short telencephalon and short olfactory tracts are almost identical. The only major difference is the presence of a pineal recess in *Shuyu*, although in *Romundina* a shallow depression on the visceral surface of the dorsal side of the rostronasal capsule figured by Ørvig is likely to correspond to this structure ([7]:pl. 5 fig 3). The similarity extends to the position of the brain relative to the other major structures of the head. In galeaspids (and osteostracans), like in *Romundina* and *Brindabellaspis* (and antiarchs, judging by the preserved dermal skeleton), the skeleton of the face and upper lip extends far anterior to the endocranial cavity. This very close morphological correspondence suggests the somewhat surprising possibility that the shape of the anterior end of the brain and the geometrical relationship of the placode-derived structures in this region—the nasal sacs, eyes and hypophysis—remained essentially unchanged across the transition from jawless to jawed vertebrates.

A comparison of head development in lampreys and jawed vertebrates shows that the anterior part of the head is constructed from the same neural crest cell populations (supraoptic, infraoptic, mandibular) in both, but that the behaviour of these populations differs greatly between the two [21]. In lampreys, where the nasal sacs and hypophysis form from a single placode and are thus bound together throughout development, the infraoptic crest cell streams meet below the hypophysis to form the upper lip; this in turn pushes the hypophysial duct dorsally, producing the characteristic dorsally located nasohypophysial opening [21]. Mandibular crest cells form the velum and lower lip. Hagfish development appears broadly similar except that the nasohypophysial sac breaks through into the pharynx posteriorly [21, 66]. In gnathostomes, on the other hand, infraoptic neural crest cells migrate in between the hypophysial and nasal placodes, creating the trabecular-ethmoid region and leaving space for mandibular crest to move forward and build the upper jaw [21]. As a median nasohypophysial opening is widely distributed in the ostracoderms of the gnathostome stem-group, and jaws are invariably absent [67], substantial parts of the lamprey head development program including the migratory pattern of the infraoptic and mandibular neural crest can be assumed to be ancestral relative to the jawed vertebrate program. However, it should be noted that widely separated nasal sacs (which suggest separate nasal and hypophysial placodes) are present not only in galeaspids but probably also in a less crownward group, the heterostracans [67], and possibly in the pituriaspids [68]; the lamprey condition of a single nasohypophysial placode may thus not have been present throughout the ostracoderms, and may indeed not represent the primitive condition for vertebrates. In some thelodonts, there are indications of possible paired nasal sacs (see [69] and references therein for review and discussion). It is noteworthy that in *Loganellia* a series of minute anteriorly directed denticles located below the posteriorly directed dermal (superficial) denticles may indicated an anteriorly open unpaired nasohypophysial duct, quite comparable to what is observed in galeaspids [5, 70].

Notwithstanding some uncertainty about the details, it is clear that a major reorganization of the neural crest migration pattern can be mapped to the internode of vertebrate phylogeny that separates the most crownward ostracoderms from the least crownward placoderms. In *Shuyu* a dorsally oriented nasohypophysial opening is still present [19], suggesting that infraoptic ectomesenchyme migration was substantially ventral to the hypophysial placode and that the connection between the hypophysis and buccal cavity was achieved by a secondary breakthrough somewhat as in hagfish. However, a small anteriorly directed rod that projects from the rear wall of the nasohypophysial duct between the hypophysis and olfactory bulbs in *Shuyu* has been interpreted as representing the beginning of the gnathostome-style ectomesenchyme migration between these placodes to form a trabecular region [19]. By contrast, the skeleton of the premedian-ethmoid and orbital regions of *Romundina* shows characteristic gnathostome architecture: there is no nasohypophysial duct, a solid horizontal plate of endoskeleton separates the hypophysial fenestra ventrally from the nasal sacs dorsally, the nasal sacs are separated in the midline by a septum [7], and separate palatoquadrates articulate with the lateral margins of the neurocranium. This implies a gnathostome developmental program, where the infraoptic ectomesenchyme migrates forward between the hypophysial and nasal placodes to generate structures such as trabeculae, ethmoid floor and nasal septum, and is followed by mandibular ectomesenchyme on a more lateral trajectory [21]. Nevertheless, *Romundina* (like *Brindabellaspis* and antiarchs [1, 25, 61]) resembles lampreys, osteostracans and galeaspids, and differs from living gnathostomes, in having an upper lip that protrudes far anterior to the nasal sacs. This suggests that the degree of anterior proliferation of the infraoptic ectomesenchyme, as opposed to its migratory path, was conserved across the initial stage of the transition from jawless to jawed architecture. The migration and proliferation of the supraoptic neural crest seem to have been similar to those in ostracoderms, as the geometric relationship of eyes and nasal sacs is almost unchanged.

The modern gnathostome morphology, with anteriorly located nasal sacs and a posteroventral hypophysis, may likely have evolved from this initial condition by an increase in the proliferation of the supraoptic ectomesenchyme (which contributes substantially to the structures that separate the eyes and nasal sacs dorsally) coupled with an enlargement of the forebrain and lengthening of the olfactory tracts. In arthrodire placoderms such as *Kujdanowiaspis* [11, 15, 27] or *Dicksonosteus* [26, 71], the nasal sacs are located at the tip of the snout and are markedly more anterior than the hypophysis, even though the olfactory tracts are still very short. This condition may represent a step in the evolution of the modern morphology, consistent with the hypothesis that arthrodires occupy a more crownward position in the gnathostome stem than *Romundina* or *Brindabellaspis*. However, the occurrence of very long olfactory tracts in macropetalichthyid placoderms, which otherwise display many primitive neurocranial characters [1, 11, 15], suggests significant homoplasy in the evolution of these structures and cautions against simplistic scenarios. Such opposite patterns are also encountered in jawless forms (see Amphiaspididae and Cyathaspididae Heterostraci; [72]).

Another striking feature of 'posterior nose' placoderms such as *Brindabellaspis* and antiarchs (judging by the geometry of the dermal skeleton and palatoquadrates [47, 61, 73]), with potential bearing on the origin of jawed vertebrate architecture, is the remarkably anterior position of the pharyngeal arches in these animals. In *Brindabellaspis* the canal for the hyomandibular branch of nerve VII runs anteroventrally to emerge from the braincase below the anterior part of the orbit, at the same anteroposterior level as the nasal sac [25]. Antiarchs appear to have had similar proportions [61]. *Romundina* is considerably less extreme in this respect, as the corresponding nerve foramen lies below the posterior margin of the orbit. However, the differences between the two are purely proportional, not topological, and amount to the anterior postorbital process and associated structures being displaced posteriorly in *Romundina* relative to *Brindabellaspis*. According to Young's interpretation, the elements in *Brindabellaspis* are shifted forwards [25]; in this respect, *Brindabellaspis* is considered as a special condition relative to *Romundina*. In *Kujdanowiaspis* the same process is again displaced posteriorly relative to *Romundina* [11], still without any major reconfiguration of the associated vessels and nerve tracts (the facial nerve lies behind the posterior orbital wall in *Kujdanowiaspis* and other arthrodires [11, 15, 26, 27, 71], while in *Romundina* it lies in the orbit against the wall; in *Kujdanowiaspis* and *Romundina*, the hyomandibular branch of the facial nerve exits through the large anterior postorbital process, while in Phlyctaenidoidei, this branch exits just behind the slender anterior postorbital process). By contrast, in derived arthrodires such as *Tapinosteus* [11] and crown-group gnathostomes such as the early chondrichthyan *Cladodoides* [39], this association of structures—a lateral process of the braincase that is pierced by the canal for the hyomandibular branch of nerve VII, carries the epihyal artery on its ventral face, and bears articular facets for hyoid arch elements on the posterior face of its tip—is deconstructed into separate elements. Even when the orbit is bounded posteriorly by a strong lateral process, as in *Cladodoides*, this structure cannot straightforwardly be homologized with the anterior postorbital process of *Brindabellaspis*, *Romundina* and *Kujdanowiaspis*. However, the recently described stem gnathostome *Janusiscus* allows an homogenization between endocranial processes terminologies [74].

As we have seen, the presence of palatoquadrate and hyoid arch articulations either side of the hyomandibular nerve exit in *Romundina* maps the hyoid-mandibular arch boundary very precisely to the tip of the anterior postorbital process. It appears that this spatial relationship is conserved among those placoderms that possess such a process, but that the entire complex can be shifted along the antero-posterior axis. The significance of these positional differences in relation to the question of jawed vertebrate origins is difficult to assess because of the variable conditions encountered in the ostracoderm outgroups. Osteostracans have extremely anterior pharyngeal arches: the most anterior gill pouch (probably lying between the mandibular and hyoid arches) is situated well in front of the orbits [37, 75]. If this is taken as the outgroup condition for placoderms, the morphology of *Brindabellaspis* will likely be interpreted as primitive relative to that of *Romundina* and *Kujdanowiaspis*. Alternatively, the condition displayed by the Osteostraci and *Brindabellaspis* could be considered as an evolutionary convergence; *Brindabellaspis* is in many respects a very peculiar and most probably specialized form and it is not clear that the anterior position of its pharyngeal arches really represents the same condition as in osteostracans. In galeaspids such as *Shuyu* [19] the corresponding gill pouch lies behind the orbit, in a position broadly similar to the anterior postorbital process of *Romundina*. The same appears to be true in heterostracans, judging by the impressions on the internal surface of the dermal skeleton [76]. If this is used as the outgroup condition, *Brindabellaspis* will be interpreted as derived relative to *Romundina*. For the present the polarity of this interesting character must remain unresolved.

### The premedian plate: An element preserved in more derived placoderms as a vestigial element

A big premedian dermal plate is encountered in the most basal placoderms, such as the Antiarchi, some Acanthothoraci and *Brindabellaspis*. It is unknown in Petalichthyida and Ptyctodontida. This plate has never been recognized in more derived placoderms, such as the arthrodires, but a relatively small one was identified in *Entelognathus* ([3]:fig 3). This leads us to have a new look at arthrodire material.

A premedian plate is defined by either or both its location on the neurocranium and the presence of a sensory line groove or canal. An internasal plate is part of the rostro-nasal capsule. Both elements are present only in the Antiarchi (e.g. [77]:figs 4–5, PrM for premedian and aop for anterior ornamented process of the rostral plate equivalent to the internasal). The connection of the element labelled as a premedian plate in *Entelognathus* to either the neurocranium or the rostronasal capsule is uncertain. This leads us to question the element considered by Zhu *et al*. as a true premedian plate in *Entelognathus* and to consider it alternatively as an internasal plate separated from the rostral plate. This leads to the following discussion, notably when the considered element is small or poorly preserved.

The snout of basal Arthrodira (Actinolepidoidei and Phlyctaenii) and basal Brachythoraci is reasonably well known in a number of forms.

In *Kujdanowiaspis buczaczensis*, a small dermal and tuberculated element is observed in the midline on the ventral side of the snout between the exhalant external nares and posteriorly to the ventral lamina of the rostral plate. This element was named a prerostral plate by Stensiö (Ra, [48]:figs 4–6; [11]:fig 10A) who noted that it was intimately connected with the rostral plate although he could not identify the boundary between these two plates. Stensiö also surprisingly considered it as a component of the postnasal plates, but Goujet showed that this was incorrect, and considered that this element was in fact a process of the rostral plate [26]. However, Dupret ([27]:13) considered there was not enough evidence to determine whether it was a posterior protrusion of the rostral plate or a distinct element such as the internasal plate of *Coccosteus cuspidatus* [78].

In *Buchanosteus sp*., a distinct dermal bone is located ventrally between the two nares and named internasal bone, as for its homologue in *K*. *buczaczensis*; the internasal bone covers the internasal wall (respectively IN, iw, *in* [79]:figs 103–104). However, we believe that Miles wrongly labelled the most posteroventral portion of the rostral plate as internasal in his figure 105. This terminology was followed by Young [80], who attributed the species to *B*. *confertituberculatus* [81].

Owing to the light of recent work [6], we reinterpret this median dermal element in *Kujdanowiaspis* and *Buchanosteus* as a true although vestigial premedian plate. A reduction of the anterior trabecular area coupled with a rotation of the external nares from facing anteriorly to ventrally would have resulted in the shrinkage and ventral internasal location of the premedian plate, together with its dislocation from the neurocranium and the loss of a sensory line groove.

One could object that this element is not present in all Arthrodira. However, in *Dicksonosteus arcticus*, a bony septum is identified between the internasal wall ("cloison nasale") and the ventral internasal lamina of the postethmo-occipital bone on the ventral side of the neurocranium (respectively m.i and *l*.*i*.*v in* [26]:54; Fig 8), and ensures the connection between the rostronasal capsule to the rest of the skull (character 1 in [82]). This bony septum is independent from the rostral plate, and is located in the same position as *Kujdanowiaspis buczaczensis* or *Buchanosteus confertituberculatus*. But contrary to these two taxa, the ventral side of the neurocranium in *Dicksonosteus* protrudes a median internasal lamina. This lamina would have prevented the formation of a proper dermal bony element with external tubercles. We hypothesize that the premedian plate is lost in *Dicksonosteus*, and that the internal bony septum represents in fact the optic fissure, separating the rostronasal capsule from the postethmoid component of the neurocranium (as in [26]).

Finally, it is noteworthy that the presence of a premedian plate is supposed in an undescribed groenlandaspidid from Australia (Ritchie, pers. com.; Dupret et al., in prep.).

### The hypophysial duct

The hypophysis displays two main patterns in extant vertebrates: in cyclostomes, it opens into a nasohypophysial duct that opens onto the face anteriorly, whereas in gnathostomes the hypophysial duct (which lacks any connection with the nasal sacs) opens onto the palate. It appears that fossil forms illustrate a series of steps in the shift of this structure. In the jawless galeaspid *Shuyu*, the hypophysial duct is oriented anteriorly in the naso-oral cavity [19]. In the basal placoderms *Romundina* and *Brindabellaspis*, the duct is directed anteroventrally and opens in the mouth. It is noteworthy that in arthrodire placoderms, such as *Kujdanowiaspis*, *Dicksonosteus* and *Buchanosteus* [11, 15, 26, 27, 71, 83, 84], the duct is rather short, but also directed ventrally, An hypophysis positioned posteriorly and further apart from the nasal sacs occurs in the crown-gnathostome clade [6, 21, 66, 85–88].

### Presence of a parasphenoid

The imaging of specimen MNHN.F.CPW1 reveals the presence of material "plugging" the hypophyseal fenestra. This material does not resemble dermal bone, and is not consistent with the surrounding limestone matrix. It is however reminiscent of the structure of the matrix which was partially dissolved during acid preparation. Nevertheless, as the vacuities of the structure situated in the hypophyseal window were reconstructed, they revealed a three-dimensional pattern similar to that of a vascular mesh, which could correspond to that of a parasphenoid (Fig 14). A pair of symmetric anteroposteriorly directed canals is visible on the internal (dorsal) side (arrows, Fig 14); a few transverse canals are visible too, although incomplete and more slender. Because of this mesh-like organisation, we consider the presence of a parasphenoid element plausible in *Romundina*. Additionally, the ventral face of the neurocranium also displays a shallow rim around the hypophyseal window, which could probably correspond to the flanges of the parasphenoid.

The placoderm parasphenoid is usually a bone of mainly perichondral nature, although covered by tubercles on its palatal side in some forms and possibly thick in some forms (e.g. Brachythoraci). It penetrates deeply the neurocranium to fill the whole hypophyseal cavity.

There is no evidence of such a bone in other specimens of *Romundina*. It is noteworthy that Antiarchi (except for one specimen of *Bothriolepis* from the Gogo formation in Western Australia; [60]:47), Petalichthyida and Ptyctodontida lack a parasphenoid, while the order Arthrodira displays various shapes [89]. It is also absent in chondrichthyans and Acanthodians [5].

The presence of a parasphenoid in other "Acanthothoraci" is not consistent throughout this group. It is known as a very elongate element in *Kosoraspis peckai* [90], but absent (or not preserved) in *Radotina kosorensis* [90, 91].

If the presence of parasphenoid is considered as a reliable phylogenetic character that appeared only once, it would mean that taxa devoid of it display the primitive condition. In that case, this would support a basal position for the Ptyctodontida (see [1, 92], where Ptyctodontida are sister-group with Petalichthyida), rather than a derived one (sister-group with *Entelognathus* and or crown-ganthostomes; see [3]).

*Romundina* being in a phylogenetic position intermediate between Antiarchi and Arthrodira [6], it is possible that we face an intermediate condition between a lack of parasphenoid and a neatly defined and shaped bone one (i.e. non completely ossified).

### The endolymphatic duct complex

The endolymphatic duct (d.end) is a tube linking the sacculus of the inner ear and the external environment via the endolymphatic pore. In extant chondrichthyans, this duct is filled with seawater. Its precise function is still poorly understood, although some tend to think it could be linked with audition. The duct "has been hypothesized to act as a site of release of displacement waves" [93], as any flow induced over the fenestrae ovalis would propagate down the posterior canal duct and into the sacculus. Also, the sacculus, lagena and utriculus are thought to be involved in both balance and sound reception. They are composed of sensory hair cells on an epithelium overlain by an otoconial mass (otolites in a mucous substance), which acts as an inertial mass. Some taxa even add exogenous material (e.g. sand grains in *Squalus acanthias*) in order to increase the ability of their endogenous otoconial mass [93–95].

The shape of the endolymphatic duct is very variable among the taxa. Extant sharks show a vertical tube opening on the external environment through a single common endolymphatic pore (e.g. *Squalus acanthias*, see [96]). Rays exhibit a more complex trajectory: the pore opens in a short tube connected to a horizontal endolymphatic sac, the latter being connected to the endolymphatic tube linked to the sacculus (e.g. *Raja clavata*, see [97]).

*Brindabellaspis* is the only placoderm in which an endolymphatic sac has been identified. This sac is horizontal and situated just posteriorly and above the saccula and below in the endolymphatic duct (see [25]:text-figs 10, 12). The endolymphatic duct itself is subvertical and oblique, and is connected to a transversally oriented pocket just below the dermal skull roof (another sac?) open to the external environment via a tiny pore. The endolymphatic pores opens just laterally to the midline on the nuchal plate.

In antiarchs the endolymphatic ducts are known only in *Minicrania* (see [98]:4; Figs 3A, 6 and 7), and extend posteromedially from a paired swelling that may have contained endolymphatic sacs. The pores open in the midline on the nuchal plate.

In Petalichtyida where it is known, the endolymphatic duct is simple and vertical. The pore opens on the anterior paranuchal plate. In basal Petalichthyida such as *Eurycaraspis*, the presence of an external pore is disputed ([99] vs. [100] on *Eurycaraspis*. The absence of an external pore (i.e. possibly a blind duct), allied to the basal position of *Eurycaraspis* among Petalichthyida pledges for a close relationships between Petalichthyida and Ptyctodontida, the later being equally devoid of endolymphatic pore; this sister-group relationship is proposed by Goujet and Young [101] but is not recovered with most recent phylogenies (where *Macropetalichthys* is sister-group of *Brindabellaspis*, and Ptyctodontida are sister group of Acanthodians and crown-gnathostomes [2, 3, 6]; Brazeau resolves *Macropetalichthys* as sister-group of Ptyctodontida, this clade being more coronal than *Brindabellaspis* but more basal than Arthrodira [1]).

In Arthrodira, the endolymphatic duct is a long oblique and straight tube that exits from the endocranium and continues within the paranuchal plate. It opens externally through an endolymphatic pore situated on the paranuchal plate. The position of this pore on the plate differs among subgroups: although always situated at the radiation centre of the plate, it is located at the geometric centre in Actinolepidoidei (see for example *Kujdanowiaspis podolica*; [15]:fig 43; [27]:fig 6), while it is closer to the posterior edge in Phlyctaenioidei (Phlyctaenii and Brachythoraci; see for example *Dicksonosteus*; [26]:fig 4). It is noteworthy that the size of the duct is unchanged despite the position of the external foramen; this thus reflects a different length of the occipital parts of the neurocranium.

The endolymphatic duct of *Romundina* is straight and very slightly oblique. The lumen seems larger, and the walls of the canal seem thicker than what is observed in other forms. The external endolymphatic foramen opens at the level of the geometric centre of the posterior paranuchal plate. The endolymphatic canal crosses the canals for the vagus and glossopharyngeal nerves medially from the saculla of the inner ear, in a condition similar to that observed in *Kujdanowiaspis*, but different from that of *Dicksonosteus* [15, 26]. In all placoderm taxa where it is preserved, the anteriormost portion of the endolymphatic duct shows a sub-horizontal orientation. On the contrary, chondrichthyans show a consistent vertical trajectory all along.

From an embryological point of view, as the otic placode sinking occurs very early in development, the length, direction and shape of the endolymphatic duct provide information about the dynamics of the tissues surrounding the duct and its external opening.

It is noteworthy that the endolymphatic duct of *Romundina* is paralleled by a ventral slender canal, most likely vascular and connected to the dermal bone (arrow, Fig 10C and 10D). The duct itself is surrounded by a vascular sheath connected to the dermal bone. Because of their disposition, we believe that the formation of this slender canal and of the endolymphatic duct are synchronous. This indicates that the vasculature (or at least the main vessels irrigating the embryo) was already individualizing (if not already established) when the otic placode sank. This is reminiscent of what was described of the vascular mesh development of premedian plate of *Romundina* [13].

In a similar way, the vertical canals that connect the jugular vein canal to the underside of the dermal skull roof, and which curve around the saccular region of the inner ear in such a manner that they maintain an approximately constant distance to its surface, may give an indication of the kinematics of the shaping of the otic area.

### The notochord

Although obviously not preserved, it is possible to reconstruct the notochord, using the aspect of the natural surface of the perichondral bone covering the ventral side of the neurocranium and the perichondral bony floor of the endocranial cavity (*dura mater* of [11]).

Due to compression and diagenesis, the posterior part appears lower than the most anterior part. The notochord can unfortunately be reconstructed only until the oblique crack, in the otic area (at the level of the glossopharyngeal nerve IX). Nevertheless, the width of the groove on the ventral side of the endocranial cavity suggests a more anterior extension. As a matter of fact, at the level of the pituitary vein canal, a slender and short longitudinal groove is visible; but this groove is very short and obscured by the oblique crack. Hence we cannot determine with certainty whether this groove corresponds to the most anterior extension of the notochord or not.

In many cases where the fossil material has not been chemically prepared or serially ground, the length of the notochord can only be estimated by means of the chordal ridge visible on the ventral side of the neurocranium.

There is no preserved specimen of *Kujdanowiaspis* showing the ventral side of the neurocranium, and we have to rely entirely on Stensiö’s reconstructions, although with caution. In *Kujdanowiaspis*, he reconstructed the chordal ridge extending forwards to a level between the anterior and posterior postorbital processes ([11]:fig 10B; see also [26]:fig 9; [27]:fig 5). However, only one specimen of *Kujdanowiaspis podolica* (known only by one photograph; see [27]:fig 11) exhibits a chordal groove (a mould of the neurocranial floor is visible) extending at least up to the level of the spino-occipital nerve. More anteriorly, the ventral floor of the neurocranium is covered by the infilling of the endocranial cavity; however, given the material at disposition, a more anterior extension cannot be excluded. In *Heightingtonaspis* (a form closely related to *Kujdanowiaspis*; see [82, 102–105]), the chordal ridge extends anteriorly to the level of the posterior branch of the postorbital process; it is uncertain whether this structure extends more anteriorly in the neurocranium (see [27]:fig 10).

Stensiö, using grinding series of *Kujdanowiaspis rectiformis* (i.e. most probably *K*. *podolica* or *K*. sp.; see [27] for taxonomic considerations), illustrated the chordal canal, although in dashed lines because this canal is not preserved *per se* ([11]:fig 29C, 31–32, 35). Indeed, Stensiö [11] specifies in the caption of fig 31 only that the canal is “not preserved”. Anyhow, the most anterior “occurrence” of this chordal canal in *Kujdanowiaspis* is illustrated at transverse section number 295, that is at a position “somewhat caudal to the space of the ampulla anterior”; the previous illustrated section—number 290—does not show the chordal canal, and is at a position “showing the caudal part of the anterior postorbital process” ([11]: caption of fig 29) As well, he labels this structure either as “chordal canal” or as “space for the notochord”. As such, the chordal canal would extend only little further anteriorly than the chordal ridge illustrated in his fig 41. However, Stensiö extended the notochord as far as just anteriorly to the pituitary vein in his hypothetic reconstructions of “embryonic cartilages of the basal parts of the adult *Kujdanowiaspis*-endocranium” ([11]:fig 22A, C). According to one of us (D.G.), such an anterior extension of the chordal canal is related to the size of the pituitary vein canal: because of its width, Stensiö considered it also included the rectus externalis ocular muscle, a pattern also encountered in actinopterygians in which the muscle is posteriorly extended until an attachment in the chordal canal. This pattern is however improbable for placoderms (lack of evidence, despite the size of the pituitary vein canal in some forms; see [26]:65–66), but it nevertheless reflects the author’s research of an archetypal, ancestral feature, and the transposition in his descriptions of elements encountered in other forms as long as those latter comfort his interpretations.

It is noteworthy that Stensiö also reconstructed the chordal canal of *Tapinosteus heintzi* as far anteriorly as transverse section number 421 (“section caudal to the facialis canal”; [11]:fig 61A); but the canal is reconstructed in dashed lines as is the case in *Kujdanowiaspis*. However, anteriorly to this section, the specimen he used was badly preserved ventrally and some information lacks in the midline area (e.g. see [11]:fig 61–62). This was not the case of the specimen of *Kujdanowiaspis*, but one can admit that the endocranial cavity is collapsed (e.g. see [11]:fig 27). Because Stensiö reconstructed a chordal canal he actually did not see but only suspected (his technicians drew what they observed very minutiously, and Stensiö added the canal afterwards; DG pers. obs.), we can only wonder how far this canal extended in reality in *Kudjanowiaspis*: up to the pituitary vein like in his embryonic cartilage reconstruction, or up to the level of the anterior postorbital canal.

In the acid prepared *Brindabellaspis*, Young ([25]:text-fig 7, 11) illustrates the anterior extension of the chordal ridge until the level of the large posterior branch of the vagus nerve X (that also corresponds to the position of the posterior postorbital process). This is a rather posterior position for the tip of the notochord.

Stensiö ([14, 15]:fig 49, 50B) illustrates in *Macropetalichthys rapheidolabis*? a chordal groove extending up to the vagus nerve level (that is all along the narrow occipital part of the neurocranium), then transforming into a chordal ridge up to the level of the hypophysial foramen. This anterior extension is consistent with what we observe in our specimen of *Romundina*.

In *Kosoraspis peckai* [90] (see [15]:fig 9A), one can also notice a posterior groove followed by an anterior chordal ridge up to the hypophysial foramen, although the ridge seems wider in *Kosoraspis* than in *Macropetalichthys*. In *Radotina kosorensis* (see [15]) there is a ridge from the posterior edge of the neurocranium unto the level of the anterior postorbital process, which is situated much posteriorly behind the hypophysial fenestra. None the less, there is no certainty as to the extension of the chordal ridge and groove represent the real extension of the overlying notochord.

A groove in the posterior part of the occipital area of the neurocranium (below the chordal ridge, or at its level) is correlated with the presence of an unpaired canal of the dorsal aorta (as hypothesized by [25]:fig.26A), this feature itself being related to an elongation of the occipital region of the neurocranium (see [106]:585), contrary to short occipital forms like *Kujdanowiaspis*, *Heightingtonaspis* and *Dicksonosteus* (see respectively [11]:fig.41; [15]:fig 57; [27]:fig 10; [26]:fig 51) where the dorsal aorta tract is paired (i.e. the confluence occurs behind the occipital condyles). Nevertheless, in specimen MNHN.F.CPW1 of *Romundina stellina*, although this area of the braincase is poorly preserved, it is possible to estimate the position of the dorsal aorta confluence with the laterodorsal aorta tracts, using the prolongation in the reconstructed fragments of the vessel; such confluence would occur in the middle of the otic area, i.e. between nerves IX and X, a pattern existing in two other specimens of *Romundina* from the same lot in the Paris collection. This is ascertained by some other specimens that came to our knowledge after segmentation of CPW1 was processed (MNHN.F.CPW6 and CPW2a-b, S1 and S2 Figs).

Interestingly, a shallow depression is visible on the ventral side of the neurocranium of *Kosoraspis peckai* and *Radotina kosorensis*, approximately between nerves IX and X. This depression is diamond shaped in *R*. *kosorensis* and looks like a reverse drop in *K*. *peckai*. Such a depression is also visible in *Macropetalichthys*, although much smaller and more slender. Although this could be related to diagenesis and compaction in a fragile area of the neurocranium, a similar depression is visible on the ventral side of the filling of the endocranial cavity of *Romundina stellina*; this one is situated at the level and anteriorly from nerve X exit, its most anterior extension remaining unknown because of the oblique crack. We have no idea whether this depression is indicative of a special function or a phylogenetic signal.

### The “lace pattern” between the trigeminal and vagus nerves

We have shown earlier that the perichondral lace pattern observed between the trigeminal and vagus nerves is original, and as such is not the consequence of bad preservation or microbial activity, given both its precise location and the observed symmetry in the ossified and non-ossified areas. Nevertheless, the exact nature of this localised lace pattern remains to be explained.

P. Janvier (com. pers.) suggested that the thickness (and consistency) of the perichondral bone could be related to the distance between perichondral and dermal bones, or to the anterior extension of the notochord. Nevertheless, an accurate analysis of the distance between the dermal and perichondral bone of the internal structures along the antero-posterior axis does not reveal such a correlation with the thickness of the dermal bone (Figs 4, 5 and 15; S1 Video). On the contrary, a short or long distance between internal perichondral and dermal (with perichondral lining) bones displays both patterns (lacy or not). More endocranial material corresponding to specimens at various ontogenetic stages and phylogenetic positions would be necessary to clarify this point (study in progress).

## Conclusion

The skull of a specimen of *Romundina stellina*, a basal placoderm from the early Devonian of the Canadian Arctic Archipelago, was scanned at the European Synchrotron Radiation Facility using propagation phase contrast synchrotron microtomography, in order to reveal its internal structures. The combination of a non-destructive data acquisition technique and a segmentation process that works directly with the scan data set allows us to provide a virtual model of the intracranial structures that is more objective and less likely to be affected by interpretative bias than the grinding-series based wax models initiated by Sollas [107] and developed by the Stockholm school under Stensiö's direction [55]. The digital 3D reconstruction provides fundamental information regarding the central nervous system and cranial nerves, including the sensory line system, the vasculature with distinguished veins and arteries, the inner ear, the jaw attachment, the notochord and a putative parasphenoid. The data missing in the scanned specimen were recovered from co-existing specimens of the same species. The basal phylogenetic position of *Romundina* within the gnathostome tree highlights the importance of such an anatomical atlas.

*Romundina* shows a mosaic of features belonging to either gnathostomes or cyclostomes. Among other organs, the inner ear is described in details for the first time. It differs from that of both osteichthyans and chondrichthyans by its general rounded shape, and the endolymphatic duct differs from that of other placoderms. Whether the condition displayed in *Romundina* can be considered as a primitive condition for jawed vertebrates and/or shared with most placoderm orders will require more fossil material where this region of the neurocranium could be studied (Long et al., work in progress). Although not preserved, the branchial arches of *Romundina* were probably seriated as in extant sharks but more anteriorly positioned under the neurocranium, instead of behind it.

This study opens new perspective in the development and pattern analysis of the bone vascularization (study in progress); it confirms several interpretations on the nerve disposition and the orbit anatomy.

The gap between extant cyclostomes and gnathostomes is enormous. Only the minute study of internal structures of jawed and jawless forms of both fossil and extant vertebrates will bring discussion material to fill in this gap. Fortunately, new technologies facilitating the acquisition and segmentation processes and new material such as more complete developmental series including fossil embryos will contribute to increase our understanding of our deep jawed vertebrate origins.

## Supporting information

S1 Fig Skull of *Romundina stellina* Ørvig, 1975. **Specimen MNHN.F.CPW6.** A. Dorsal view. B. Left lateral view. C. anterior view. D. posterior view. Scale bar 10 mm. Notice in B the light in the foramen for the pituitary vein indicating that it opens in a canal opening in the right orbit.
(TIF)

S2 Fig *Romundina* sp. Specimen MNHN.F.CPW2a-b. A. Dorsal view, MNHN.F.CPW2a. B. Ventral view, MNHN.F.CPW2b. Scale bar 10 mm. Notice in B the groove connecting the efferent and laterodorsal artery grooves (white arrows).
(TIF)

S1 Table Anatomical abbreviations used in the figures. (XLS)

S1 Text Remark concerning the myodomes and the extrinsic muscles in the orbit. (DOC)

S1 Video *Romundina stellina*, MNHN.F.CPW1, complete run-through (contrasted) (complete_runthrough_contrasted_avi.avi; 19,8 MB). ImageJ.
(AVI)

S2 Video *Romundina stellina*, MNHN.F.CPW1, rotation of the dermal bone and perichondral bone vasculature (vertical_rotation.avi; VG StudioMax, SS and PT). (AVI)

S3 Video *Romundina stellina*, MNHN.F.CPW1, apparition of the 3D model emerging from below the photograph of the actual specimen. The dermal skull roof opens to reveal the internal perichondral structures. 0001–0125 (nouveau).avi: Maya Autodesk; 3,88 MB.
(AVI)

S4 Video *Romundina stellina*, MNHN.F.CPW1, rotation around the internal perichondral bone ossification, revealing the lace pattern. 0125–400 (nouveau).avi: Maya Autodesk; 21,3 MB.
(AVI)

S5 Video *Romundina stellina*, MNHN.F.CPW1, filling of the internal structures and rotation around the specimen. (0400–0750 (Nouveau).avi: Maya Autodesk; 25 MB.
(AVI)

S6 Video *Romundina stellina*, MNHN.F.CPW1, rotation of the endocranial cavity. olaf_002: Mimics; 5,28 MB.
(AVI)

S7 Video *Romundina stellina*, MNHN.F.CPW1, rotation of model without external perichondral and dermal bone. olaf_003; Mimics;11,4 MB.
(AVI)

S8 Video *Romundina stellina*, MNHN.F.CPW1,: rotation of model with external perichondral bone opaque. olaf_004; Mimics;16,9 MB.
(AVI)

S9 Video *Romundina stellina*, MNHN.F.CPW1, rotation of model with external perichondral bone semitransparent. olaf_005; Mimics;16,9 MB.
(AVI)

S10 Video *Romundina stellina*, MNHN.F.CPW1, rotation of the right inner ear. olaf_006: Mimics; 8,61 MB.
(AVI)

S11 Video *Romundina stellina*, MNHN.F.CPW1, rotation of left inner ear. olaf_007; Mimics; 8,35 MB.
(AVI)

S12 Video *Romundina stellina*, MNHN.F.CPW1, rotation of dermal bone vasculature, nerves, arteries and veins. olaf_010; Mimics; 12,5 MB.
(AVI)

S13 Video *Romundina stellina*, MNHN.F.CPW1, rotation of opaque dermal bone of the skull roof. olaf_012; Mimics; 14 MB.
(AVI)

S14 Video *Romundina stellina*, MNHN.F.CPW1, rotation of specimen semitransparent showing inner structures. olaf_018; Mimics; 16,3 MB.
(AVI)

S15 Video *Romundina stellina*, MNHN.F.CPW1, rotation of endocranial cavity, inner ears and right endolymphatic duct. olaf_019; Mimics; 10,5 MB.
(AVI)

S16 Video *Romundina stellina*, MNHN.F.CPW1, endocranial cavity filled plus cranial nerves plus part of the sensory line system. olaf_020: Mimics; 7,73 MB.
(AVI)

S17 Video *Romundina stellina*, MNHN.F.CPW1, rotation of external perichondral bone semitransparent with internal vacularization opaque. olaf_021; Mimics; 12,1 MB.
(AVI)

S18 Video *Romundina stellina*, MNHN.F.CPW1, rotation of external perichondral bone semi-transparent, inner structures opaque (nerves, arteries, veins). olaf_024: Mimics; 63,1 MB.
(AVI)

S19 Video *Romundina stellina*, MNHN.F.CPW1, rotation of right inner ear and endolymphatic duct. olaf_030; Mimics; 60,9 MB.
(AVI)

S20 Video *Romundina stellina*, MNHN.F.CPW1, rotation of dermal and external perichondral bones semi-transparent; filled endocranial cavity and cranial nerves, sensory line system. olaf_032; Mimics; 79,6 MB.
(AVI)

S21 Video *Romundina stellina*, MNHN.F.CPW1, rotation of dermal and external perichondral bones opaque; filled endocranial cavity and cranial nerves, sensory line system. olaf_035; Mimics; 79,6 MB.
(AVI)
