# Supplementary figures and images for: The internal cranial anatomy of Romundina stellina Ørvig, 1975 (Vertebrata, Placodermi, Acanthothoraci) and the origin of jawed vertebrates—Anatomical atlas of a primitive gnathostome
Source: PLoS One. 2017 Feb 7;12(2):e0171241. doi: 10.1371/journal.pone.0171241 (PMC5295682; doi:10.1371/journal.pone.0171241)

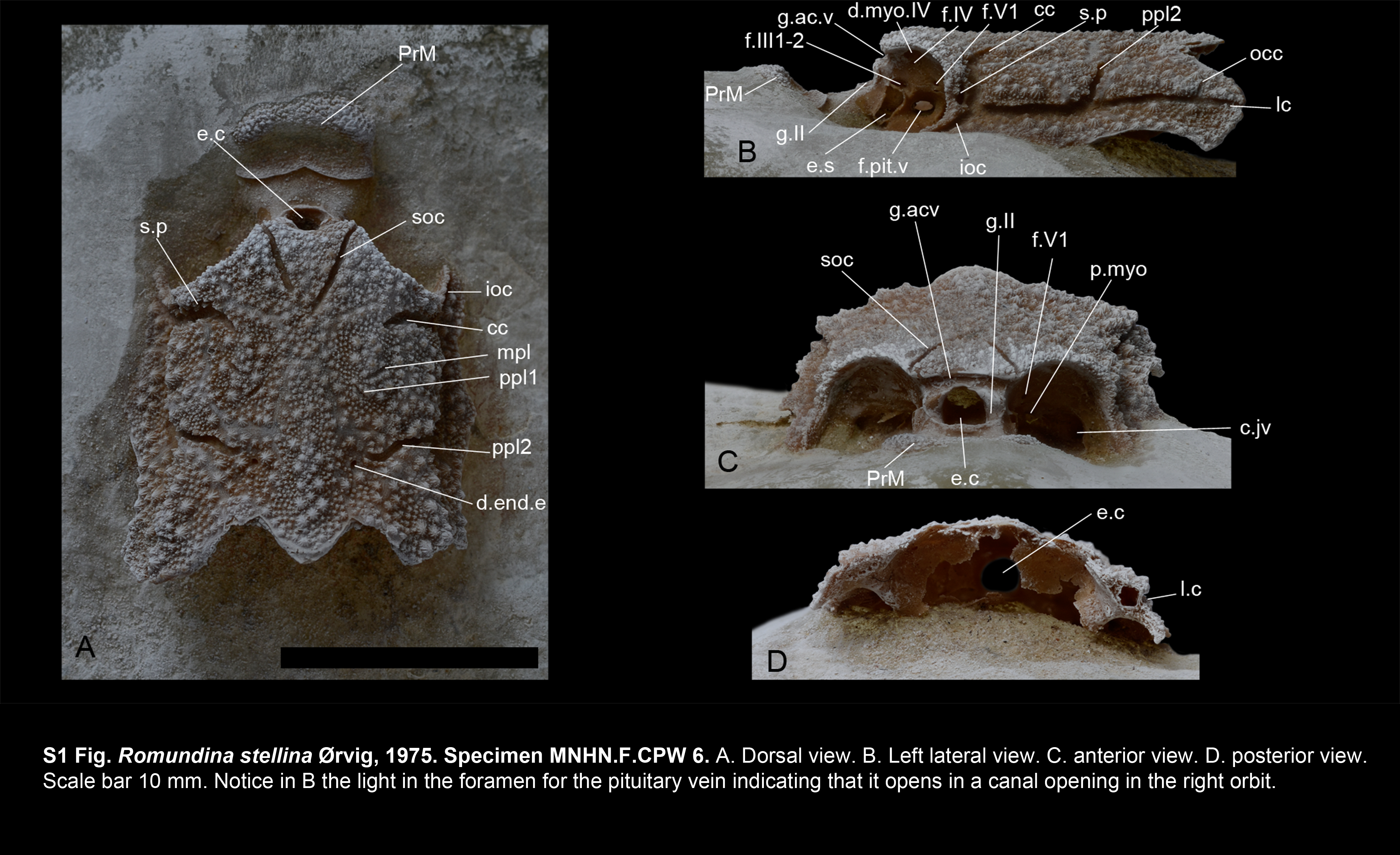

Supplement: S1 Fig — Specimen MNHN.F.CPW6. A. Dorsal view. B. Left lateral view. C. anterior view. D. posterior view. Scale bar 10 mm. Notice in B the light in the foramen for the pituitary vein indicating that it opens in a canal opening in the right orbit. (TIF) [file pone.0171241.s001.tif]

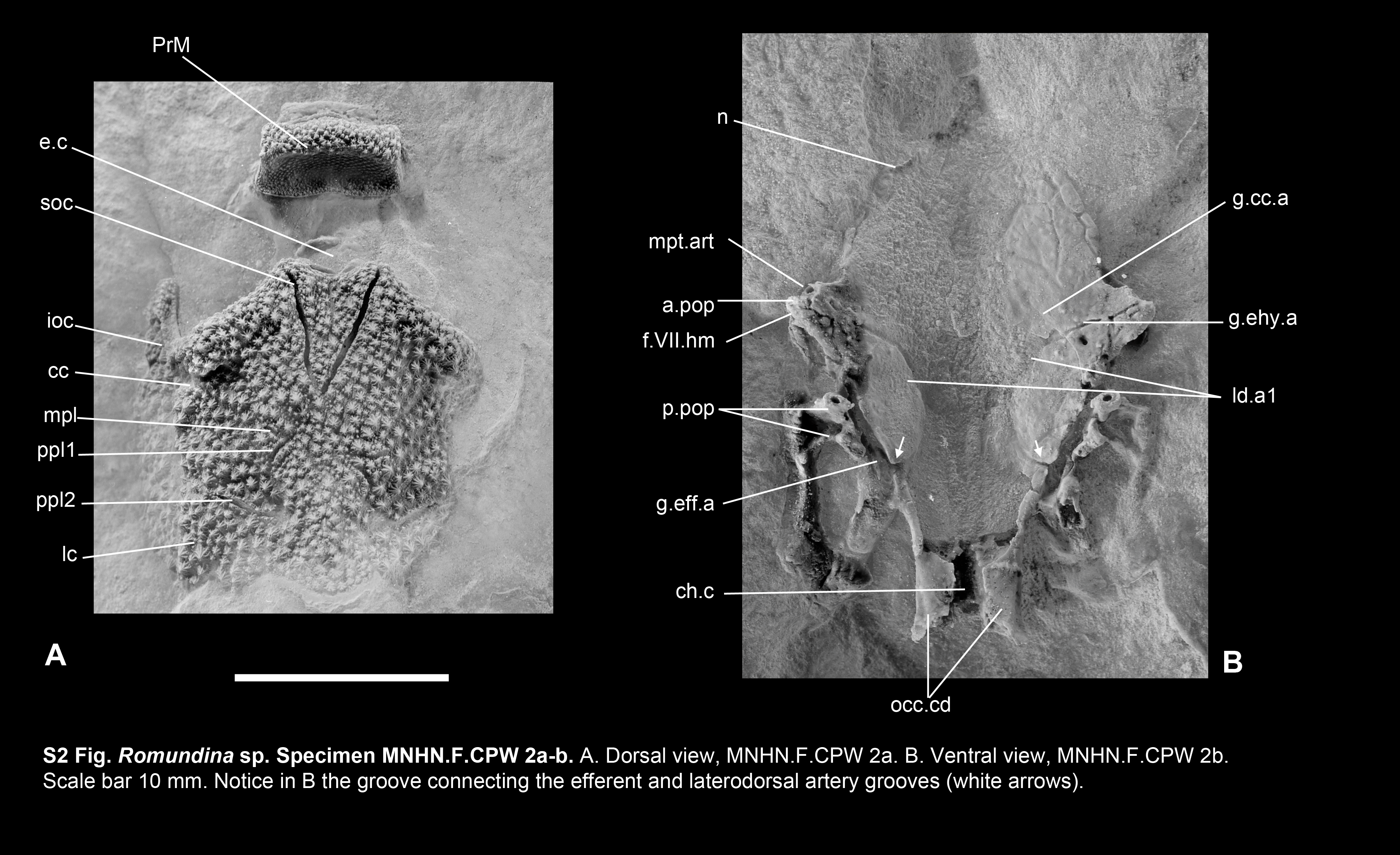

Supplement: S2 Fig — A. Dorsal view, MNHN.F.CPW2a. B. Ventral view, MNHN.F.CPW2b. Scale bar 10 mm. Notice in B the groove connecting the efferent and laterodorsal artery grooves (white arrows). (TIF) [file pone.0171241.s002.tif]
