# Supplementary material for: The internal cranial anatomy of Romundina stellina Ørvig, 1975 (Vertebrata, Placodermi, Acanthothoraci) and the origin of jawed vertebrates—Anatomical atlas of a primitive gnathostome
Source: PLoS One. 2017 Feb 7;12(2):e0171241. doi: 10.1371/journal.pone.0171241 (PMC5295682; doi:10.1371/journal.pone.0171241)
Supplement: S1 Text — (DOC) [file pone.0171241.s004.doc]

**Remark concerning the myodomes and the extrinsic muscles in the orbit**

In order to apprehend properly an environment, the eye ball has to be mobile. These movements are ensured by extrinsic muscles, attached to particular locations in the orbit. There are generally six extrinsic muscles in lampreys and placoderms, eight in chondrichthyans and tetrapods, and seven in non-tetrapod osteichthyans (see for review and suggested homologies), but some taxa can show different patterns. It is hence very difficult to establish homologies between the different muscles, and even more difficult to assess that of their attachment points in the orbit or the eyeball (i.e. myodome homologies). There are several ways of designating the myodomes in a vertebrate orbit: either by topological denominations (ventral myodome, dorsal myodome, etc.), or by numerical denomination (My1, My2, etc.), or a mixture of both. Each method has its advantages and disadvantges. The topological denomination is unfortunately inconsistent: the "ventral" myodome being sometimes ventral (see *Ligulalepis* ), sometimes anteroventral (see *Romundina* in :fig 1). The numerical way looses all sense of position of the myodomes with respect to each other or even to a single landmark (e.g. the eyestalk); it is inappropriate to employ a simple clock-wise count since some taxa lack (or have supernumerary) muscles; a properly consistent method would show taxa missing one or several myodome numbers, creating confusion for the reader (especially if dealing with incomplete fossil specimens, leading to more homology problems).

Ideally, each myodome should be denominated relating to its attached muscles (see ), but this is impossible to assess with certainty in fossil forms (and would possibly lead to confusion, or at least induce too much confidence).

It seems more reliable to relate mydomes with nerve exits, but once again, this may lead to a level of subjectivity in unusual patterns. Here we adopt a nomenclature based on both topology and innervation.
